# Metagenome-assembled genomes uncover a global brackish microbiome

**DOI:** 10.1186/s13059-015-0834-7

**Published:** 2015-12-14

**Authors:** Luisa W. Hugerth, John Larsson, Johannes Alneberg, Markus V. Lindh, Catherine Legrand, Jarone Pinhassi, Anders F. Andersson

**Affiliations:** KTH Royal Institute of Technology, Science for Life Laboratory, School of Biotechnology, Division of Gene Technology, Stockholm, Sweden; Centre for Ecology and Evolution in Microbial model Systems - EEMiS, Linnaeus University, Barlastgatan 11, SE-39182 Kalmar, Sweden

**Keywords:** Metagenome, Bacterioplankton, Ecology, Evolution, Marine, Brackish, Baltic Sea

## Abstract

**Background:**

Microbes are main drivers of biogeochemical cycles in oceans and lakes. Although the genome is a foundation for understanding the metabolism, ecology and evolution of an organism, few bacterioplankton genomes have been sequenced, partly due to difficulties in cultivating them.

**Results:**

We use automatic binning to reconstruct a large number of bacterioplankton genomes from a metagenomic time-series from the Baltic Sea, one of world’s largest brackish water bodies. These genomes represent novel species within typical freshwater and marine clades, including clades not previously sequenced. The genomes’ seasonal dynamics follow phylogenetic patterns, but with fine-grained lineage-specific variations, reflected in gene-content. Signs of streamlining are evident in most genomes, and estimated genome sizes correlate with abundance variation across filter size fractions. Comparing the genomes with globally distributed metagenomes reveals significant fragment recruitment at high sequence identity from brackish waters in North America, but little from lakes or oceans. This suggests the existence of a global brackish metacommunity whose populations diverged from freshwater and marine relatives over 100,000 years ago, long before the Baltic Sea was formed (8000 years ago). This markedly contrasts to most Baltic Sea multicellular organisms, which are locally adapted populations of freshwater or marine counterparts.

**Conclusions:**

We describe the gene content, temporal dynamics and biogeography of a large set of new bacterioplankton genomes assembled from metagenomes. We propose that brackish environments exert such strong selection that lineages adapted to them flourish globally with limited influence from surrounding aquatic communities.

**Electronic supplementary material:**

The online version of this article (doi:10.1186/s13059-015-0834-7) contains supplementary material, which is available to authorized users.

## Background

Microorganisms in aquatic environments play a crucial role in determining global fluxes of energy and turnover of elements essential to life. To understand these processes through comprehensive analyses of microbial ecology, evolution and metabolism, sequenced reference genomes of representative native prokaryotes are crucial. If these are obtained from isolates, the encoded information can be complemented by phenotypic assays and ecophysiological response experiments to provide insights into the factors that regulate the activity of these populations, in particular biogeochemical processes. However, obtaining and characterizing new pure cultures is invariably a slow process, even with recent advances in high-throughput dilution culturing approaches [1]. Most notoriously, the highly abundant, slow-growing oligotrophic lineages typical of pelagic environments [2, 3] remain severely underrepresented in current culture collections [4].

A very powerful alternative to obtain coherent data from individual lineages without cultivation or enrichment is single-cell sequencing [3, 5, 6]. This approach allows researchers to select certain targets of interest, based on, e.g., cell characteristics or genetic markers, to address specific research questions [5, 7, 8]. However, single-cell sequencing requires a highly specialized laboratory facility, and single amplified genomes (SAGs) typically have fairly low genome coverage, due to the small amount of DNA in each cell and associated whole-genome amplification biases [9].

Metagenomics offers an alternative shortcut to much of the information obtained from pure culture genome sequencing [10, 11]. The last decade’s revolution in DNA sequencing throughput and cost has provided researchers with the unprecedented possibility of obtaining sequences from thousands of genomes at a time in natural samples. However, despite vast amounts of sequence data allowing inferences on global distribution of phylogenetic lineages and metabolic potentials [11–14], many biogeochemical, ecological and evolutionary analyses require structuring data into genomes. This is critical because, while individual genes or genome fragments provide useful information on the metabolic potential of a community, in practice most biochemical transformations take place inside a cell, involving sets of genes structured in controlled pathways. Furthermore, genomes from naturally abundant microbes can function as references that allow high-quality annotations to be made in subsequent high-throughput environmental studies where otherwise a majority of sequences or peptides would remain unclassified [15–17]. Through the process of binning, contigs or scaffolds derived from the same lineage can be clustered and genomes reconstructed. The metagenome binning approach has been successfully applied to a range of environments, including aquatic ones [18–30]. Initially, approaches based on sequence composition (e.g., tetranucleotide frequencies) were successfully used to reconstruct near-complete genomes from metagenomic contigs without the use of reference genomes, but these methods can generally only discriminate down to the genus level [18, 20, 31]. More recently, coverage variation across multiple samples has been used, allowing binning down to species and sometimes strain level [24, 29, 32–35]. At the same time as genomes are reconstructed, the abundance distribution of these genomes across the samples is obtained, allowing ecological inferences. One alternative for automated and reproducible metagenomic binning is the CONCOCT (Clustering of contigs based on coverage and composition) program, which uses Gaussian mixture models to bin contigs using a combination of sequence composition and coverage across samples. CONCOCT was previously shown to give high accuracy and recall on both model and real human gut microbial communities [36].

The Baltic Sea is, in many aspects, one of the most thoroughly studied aquatic ecosystems [37]. It presents unique opportunities for obtaining novel understanding of how environmental forcing determines ecosystem structure and function, thanks to its strong gradients in salinity (north–southwest), redox (across depths) and organic and nutrient loading (from coasts to center), as well as pronounced seasonal changes in growth conditions. 16S rRNA gene-based studies have revealed prominent shifts in the microbial community composition along these dimensions [38–41]. The community composition of surface waters changes gradually along the 2000 km salinity gradient, from mainly freshwater lineages in the low salinity north to mostly marine lineages in the higher salinity southwest, and a mixture in the mesohaline central Baltic Sea [40]. The phylogenetic resolution of 16S amplicons, however, does not permit determining whether prokaryotic lineages are locally adapted freshwater and marine populations or represent distinct brackish strains. A recent Baltic Sea metagenomic study showed how a shift in genetic functional potential along the salinity gradient paralleled this phylogenetic shift in bacterial community composition [13]. However, since genes were not binned into genomes, different sets of distinguishing gene functions could not be assigned to the genomic context of specific taxa. Reference genomes would therefore be invaluable for a richer exploration of available and future omics data.

Here, we used metagenome time-series data from a sampling station in the central Baltic Sea to generate metagenome-assembled genomes (MAGs) corresponding to several of the most abundant, and mostly uncultured, lineages in this environment. We use these data to compare functional potentials between phylogenetic lineages and relate functionality with seasonal succession. By comparing the MAGs with metagenome data from globally distributed sites, we propose that these are specialized brackish populations that evolved long before the formation of the Baltic Sea and whose closest relatives are today found in other brackish environments across the globe.

## Results and discussion

### Metagenome-assembled genomes

We conducted shotgun metagenomic sequencing on 37 surface water samples collected from March to December in 2012 at the Linnaeus Microbial Observatory (LMO), 10 km east of Öland, in the central Baltic Sea. On average, 14.5 million read pairs were assembled from each sample, yielding a total of 1,443,953,143 bp across 4,094,883 contigs. In order to bin contigs into genomes, the CONCOCT software [36] was run on each assembled sample separately, using information on the contigs’ coverages across all samples (Figure S1 in Additional file 1). Single-copy genes (SCGs) were used to assess completeness and purity of the bins. We approved bins having at least 30 of 36 SCGs present (Additional file 2), of which not more than two were in multiple copies. This resulted in the identification of 83 genomic bins, hereafter referred to as metagenome assembled genomes (MAGs). The completeness of these MAGs was further validated by assessing the presence and uniqueness of a set of phylum- and class-specific SCGs (n = 119–332; detailed in Additional files 2 and 3). Based on these SCGs, we estimate the MAGs to be, on average, 82.7 % complete with 1.1 % of bases misassembled or wrongly binned, with some MAGs estimated to be 100 % complete (Table 1). In comparison, recent single amplified genome studies of free-living aquatic bacteria have obtained average completeness of 55–68 % [3, 6]. Importantly, the number of MAGs reconstructed from each sample correlates with the number of reads generated from it and there is no sign of saturation in this trend (Figure S2 in Additional file 1), meaning that more genomes can easily be reconstructed by deeper sequencing of the same samples. Every sample with over 20 million reads passing quality control yielded at least three approved genome bins. Further, while only highly complete genomes were selected for this study, other research questions might be adequately addressed with partial genomes, many more of which were generated.

**Table 1:** Overview of clusters, sorted by taxonomy

Cluster	Number of MAGs	Average bin size (Mb)	Coding (%)	GC (%)	Taxonomy	Percentage average abundance (maximum)	Percentage average completeness (maximum)
BACL2	7	1.07	94.3	44.3	*Actinobacteria*; acI	1.20 (6.47)	78.7 (88.2)
BACL4	6	1.01	94.8	41	*Actinobacteria*; acI	0.36 (1.16)	80.8 (90.4)
BACL15	2	1.11	94.9	47.1	*Actinobacteria*; acI	0.37 (1.19)	84.2 (93.4)
BACL6	4	1.55	94.8	51.3	*Actinobacteria*; acIV	0.34 (2.92)	80.0 (86.0)
BACL17	2	1.45	95.5	52.3	*Actinobacteria*; acIV	0.21 (1.10)	82.7 (93.4)
BACL19	1	1.26	95.3	58.1	*Actinobacteria*; acIV	0.09 (0.57)	59.6
BACL27	1	1.67	94.7	50.4	*Actinobacteria*; acIV	0.29 (1.73)	69.9
BACL25	1	1.26	93.5	55.7	*Actinobacteria*; Luna	0.08 (0.62)	77.2
BACL28	1	1.12	94	51.7	*Actinobacteria*; Luna	0.13 (0.96)	65.4
BACL10	3	2.57	90.2	50.5	*Alphaproteobacteria*; *Rhodobacter*	1.22 (10.91)	84.0 (88.7)
BACL5	5	1.08	96.5	30.1	*Alphaproteobacteria*; SAR11	0.79 (2.92)	80.2 (89.5)
BACL20	1	1.14	95.8	30.9	*Alphaproteobacteria*; SAR11	0.59 (2.56)	66.9
BACL7	3	1.74	95.4	49	*Bacteroidetes*; *Cryomorphaceae*	0.32 (1.40)	99.4 (100)
BACL11	3	1.19	96	32.9	*Bacteroidetes*; *Cryomorphaceae*	0.43 (1.70)	75.1 (84.9)
BACL18	2	1.32	94.5	57.5	*Bacteroidetes*; *Cryomorphaceae*	0.18 (0.93)	78.6 (85.7)
BACL23	1	1.73	95.3	54.6	*Bacteroidetes*; *Cryomorphaceae*	0.15 (1.45)	98.3
BACL8	3	1.74	93.6	38.7	*Bacteroidetes*; *Flavobacteriaceae*	0.51 (1.54)	89.4 (98.3)
BACL21	1	1.92	93.1	44.1	*Bacteroidetes*; *Flavobacteriaceae*	0.21 (1.09)	97.5
BACL22	1	2.41	91	32.1	*Bacteroidetes*; *Flavobacteriaceae*	0.14 (0.98)	91.6
BACL29	1	1.48	95.2	30	*Bacteroidetes*; *Flavobacteriaceae*	0.08 (0.49)	88.2
BACL12	2	2.64	93.6	47.2	*Bacteroidetes*; *Sphingobacteriales*	0.20 (2.14)	85.3 (93.3)
BACL14	2	1.19	94.2	38.7	*Betaproteobacteria*; OM43	0.42 (1.44)	86.5 (87.8)
BACL30	1	1.81	92.2	63.6	*Cyanobacteria*; *Cyanobium*	0.38 (1.26)	79.2
BACL3	6	2.23	90.5	53	*Gammaproteobacteria*; OM182	0.80 (3.54)	85.8 (90.9)
BACL1	14	1.37	95.2	40	*Gammaproteobacteria*; SAR86	1.74 (5.23)	85.9 (90.9)
BACL16	2	2.26	91	51.3	*Gammaproteobacteria*; SAR92	0.37 (2.41)	96.2 (97.2)
BACL26	1	1.91	91	46	*Gammaproteobacteria*; SAR92	0.13 (0.70)	83.2
BACL13	2	1.01	90.9	31.9	*Thaumarchaeota*; *Nitrosopumilaceae*	0.11 (0.88)	70.0 (78.4)
BACL9	3	1.50	93.7	56.1	*Verrucomicrobia*; LD19	0.36 (1.54)	73.6 (76.4)
BACL24	1	2.98	88.4	53.1	*Verrucomicrobia*; *Opitutaceae*	0.08 (2.25)	92.1

*BACL* Baltic Sea genome cluster

In the original CONCOCT study [36], we performed binning on a coassembly of all samples. Here we employed an alternative strategy, where binning was run on each sample separately, using the abundance profile over all samples. This way, community complexity was minimized and binning accuracy increased. Since this strategy may reconstruct the same genome multiple times over the time-series, the 83 complete MAGs were further clustered based on sequence identity. Thirty distinct clusters (Baltic Sea genome clusters [BACLs] 1–30) with >99 % intra-cluster sequence identity were formed (<70 % between-cluster identity; 95 % sequence identity is a stringent cut-off for bacterial species definition [42]), that included between one and 14 MAGs each (Table 1; Figure S3 in Additional file 1). Having several MAGs in the same cluster increases the reliability of the analyses performed, especially in the case of results based on the absence of a sequence, such as missing genes.

The genome clusters generated represent environmentally abundant strains, together corresponding to, on average, 13 % of the shotgun reads in each sample (range 4–23 %; Table 1 displays average and maximum abundance for individual genome clusters). This shows that the CONCOCT approach successfully reconstructs novel genomes of environmentally relevant bacteria.

### Phylogeny and functional potential of MAGs

**Fig. 1:**
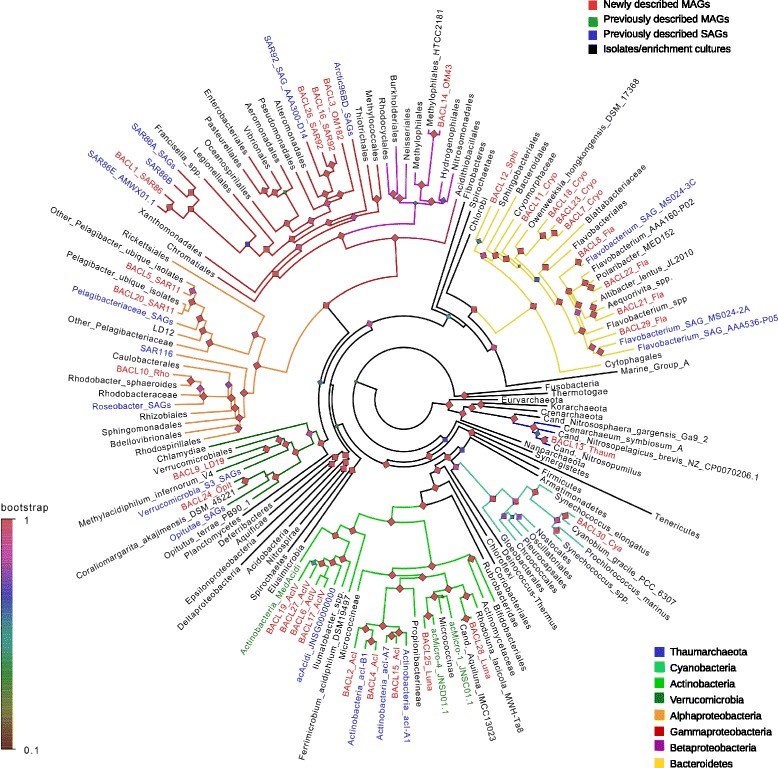
Phylogenetic tree of reconstructed genomes. In instances where several genomes were collapsed, the taxonomic label of highest resolution is displayed. Phyla and proteobacterial classes for which MAGs were generated are highlighted with colored branches. Leaf labels are colored according to the origin of the genome(s). Shimodaira-Hasegawa support for each node partition is displayed. The complete tree in newick format is available as Additional file 4 in the electronic version of this work

The reconstructed genomes belong to *Actinobacteria*, *Bacteroidetes*, *Cyanobacteria*, *Verrucomicrobia*, *Alpha-*, *Beta-* and *Gammaproteobacteria* and *Thaumarchaeota* (Table 1, Fig. 1). Phylogenetic reconstruction using >400 concatenated core proteins [43] placed all MAGs consistently with other members in their respective MAG clusters, lending further support to the binning (see Figure S4 in Additional file 1 and Additional file 4 for detailed phylogenetic trees). Based on average nucleotide identity, only BACL8 was estimated to have >70 % DNA identity with its nearest neighbor in the phylogenetic tree. In this and many other cases, the closest relative was not an isolate, but a SAG, reflecting these methods’ ability to recover genomes from abundant, but yet uncultivated, species.

**Fig. 2:**
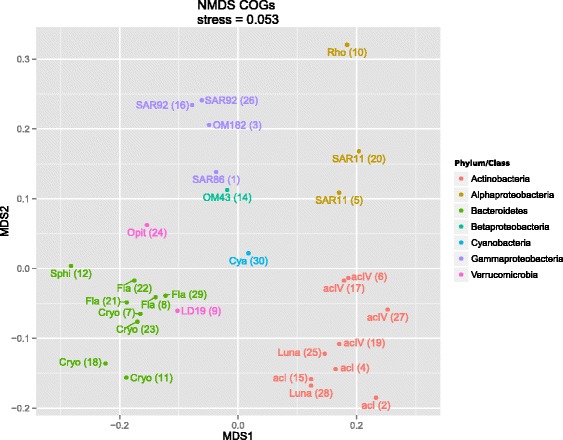
Non-metric multidimensional scaling (NMDS) of MAG clusters (BACLs) based on counts of COGs in the genomes. MAG clusters are displayed with abbreviated lineage names and BACL numbers in parentheses, and are colored according to Phyla/Class. *Cya Cyanobium*, *Cryo Cryomorphaceae*, *Fla Flavobacteriaceae*, *Opit Opitutaceae*, *Rho Rhodobacter*, *Sphi Sphingobacteriales*. The taxonomy of each BACL can also be found in Table 1

This broad phylogenetic representation allowed us to compare functional potential between taxonomic groups in this ecosystem. Non-metric multidimensional scaling based on counts of functional genes grouped the MAG clusters according to their phylogeny (Fig. 2; Figure S5 in Additional file 1; Additional file 5), which was confirmed by ANOSIM (Analysis of Similarity; Table S1 in Additional file 1). Alphaproteobacterial clusters encoded a significantly higher proportion of genes in the “amino acid transport and metabolism” COG category compared with all other clusters (Welch’s *t*-test *p* < 0.001). In contrast, *Actinobacteria* were significantly enriched in genes in the “carbohydrate transport and metabolism” COG category (*p* = 0.04), while enzymes involved in carboxylate degradation were significantly more abundant in *Gammaproteobacteria* compared with all other clusters (*p* = 0.019). Carboxylate degradation enzymes were also abundant in *Alphaproteobacteria* and *Bacteroidetes*, but significantly lower in proportion among the *Actinobacteria* (*p* < 0.01), suggesting these heterotrophs might have distinct roles in the degradation of allochthonous organic matter.

*Bacteroidetes* and *Verrucomicrobia* had the largest number of glycoside hydrolase genes, including xylanases, endochitinases and glycogen phosphorylases (Figure S6 in Additional file 1), and thus appear well suited for degradation of polysaccharides such as cellulose, chitin and glycogen, in line with previous findings connecting these groups to algal degradation [5, 27, 44]. Transporter proteins mediate many of the interactions between a cell and its surroundings, thus providing insights into an organism’s niche. A detailed analysis of transporter genes in the 30 MAG clusters is found in Figure S7 in Additional file 1 and in Additional file 6.

### Novelly sequenced lineages

The MAG approach has previously proven useful for closing gaps in the tree of life by the reconstruction of genomes from uncultivated species (e.g., [29, 30, 45, 46]). Here we report the first draft genomes for the oligotrophic marine *Gammaproteobacteria* OM182, and for the typically freshwater *Verrucomicrobia* subdivision LD19 and *Actinobacteria* clade acIV. Annotations for these genomes are found in Additional files 5 and 6.

OM182 is a globally abundant *Gammaproteobacteria* which has been grown in enrichment culture but never sequenced. BACL3 includes a 16S rRNA gene 99 % identical to that of the OM182 isolate HTCC2188 [47]. This MAG cluster shares common features with other *Gammaproteobacteria*, such as a variety of glycoside hydrolases and carboxylate degradation enzymes. It also encodes the ATP-driven sulfate transporter and a complete set of genes for assimilatory sulfate reduction to sulfide and for production of cysteine from sulfide and serine via *cysK* and *cysE*. Genes for sulfite production from both thiosulfate (via *glpE*) and taurine (via *tauD*) are also encoded in the genome, and this is the only MAG cluster to encode the full set of genes for intracellular sulfur oxidation (*dsrCEFH*). BACL3 thus appears remarkably well-suited for metabolizing different inorganic and organic sulfur sources, the latter potentially originating from phytoplankton blooms [48], even more so than previously sequenced isolates of oligotrophic marine *Gammaproteobacteria* [49].

Two verrucomicrobial genome MAG clusters were reconstructed. BACL9 MAGs include 16S rRNA genes 99 % identical to that of the globally distributed freshwater clade LD19 [50], a subdivision within the *Verrucomicrobia* still lacking cultured or sequenced representatives. Previous 16S-based analyses placed LD19 as a sister group to a subdivision with acidophilic methanotrophs [51]. Accordingly, BACL9 is placed as a sister clade to the acidophilic methanotroph *Methylacidiphilum infernorum* [52] in the genome tree (Fig. 1; Figure S4g in Additional file 1), but does not present methane monooxygenase genes and thus likely lacks the capacity for methane oxidation seen in *M. infernorum*. Interestingly, BACL9 contains a set of genes that together allow for production of 2,3-butanediol from pyruvate (via acetolactate and acetoin). Butanediol plays a role in regulating intracellular pH during fermentative anaerobic growth and biofilm formation [53]. This is also the only MAG with the genetic capacity to synthesize hopanoid lipids, which have been implicated in enhanced pH tolerance in bacteria by stabilizing cellular membranes [54]. This indicates adaptation to withstanding lowered intracellular pH such as that induced by fermentative growth under anaerobic conditions. Such conditions occur in biofilms [53], and it remains to be shown whether these planktonic bacteria can form biofilms to grow attached to particles in the water column.

BACLs 6, 17, 19 and 27 all belong to the actinobacterial order *Acidimicrobiales* and reconstructed 16S rRNA genes placed them in clade acIV. Most isolates of the order *Acidimicrobiales* are acidophilic, and no genomes have been reported for acIV, despite its numerical importance in lake water systems [55]. Previous work presented a cluster of genomes named acAcidi and tentatively placed it as an acIV [56]. However, it was at that point impossible to untangle the genomes that form the cluster, and no 16S rRNA could be assembled. Thus, the MAGs reported here are the first species-level draft genomes for this clade, and the phylogenetic tree constructed here supports the placement of cluster acAcidi as acIV (Fig. 1; Figure S4a in Additional file 1). Compared with the other typically freshwater clades acI (BACLs 2, 4, 15) and Luna (BACLs 25, 28), which belong to the order *Actinomycetales*, acIV MAG clusters have larger genome sizes and contain a significantly lower proportion of genes in the "carbohydrate transport and metabolism" COG category (*p* < 0.01), particularly ABC-type sugar transporters (Additional file 6). AcIV and acI are also impoverished for phosphotransferase (PTS) genes and amino acid transporters, compared with Luna MAGs. In contrast, acIV MAG clusters contain a significantly higher proportion of genes in the "lipid transport and metabolism" COG category (*p* = 0.02), and a significantly higher total proportion of enzymes involved in fatty-acid oxidation (*p* < 0.001), indicating that these *Actinobacteria* may use lipids as carbon source.

The only cyanobacterial genome assembled was BACL30. While it is placed in the phylogenetic tree as a distant neighbor to *Cyanobium gracile* (Fig. 1; Figure S4f in Additional file 1), its 16S rRNA gene is only 97 % identical with it, the same identity as with *Synechococcus* and *Prochlorococcus*. This genome contains genes for the pigments phycocyanin and phycoerythrin and harbors the type IIB pigment gene organization recently identified as being dominant in Baltic Sea picocyanobacteria [57]. The phycocyanin genes *cpcBA* and the intergenic spacer are 100 % identical to sequences in the type IIB pigment clade. Phylogenies of phycocyanin and phycoerythrin subunits as well as six ribosomal proteins consistently placed this cyanobacterial MAG within the type IIB pigment clades and within the clade of picocyanobacteria whose members are abundant in the Baltic Sea, but for which a reference genome has been unavailable (Fig. S8 in Additional file 1). BACL30 contains the high affinity *pstS* phosphate transporter, but lacks the *phoU* regulatory gene as well as an alkaline phosphatase. In this respect the genome is similar to the coastal strain *Synechococcus* CC9311 [58], likely reflecting adaptation to higher phosphorous loads compared with the open oceans.

### Genome streamlining and inferred cell sizes

**Fig. 3:**
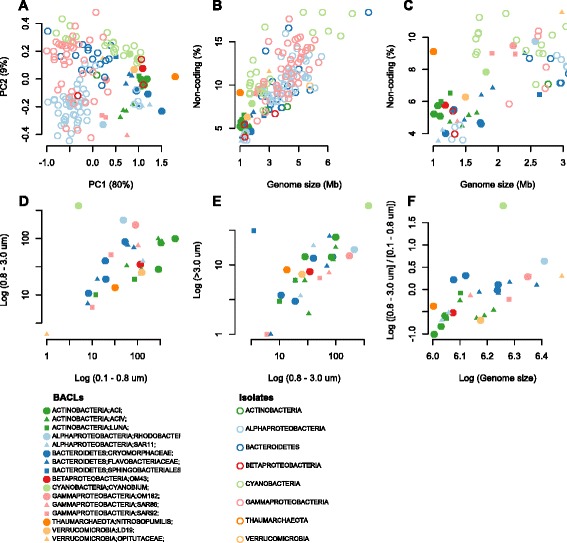
Genome properties and filter size fraction distributions of MAGs. **a** Principal components analysis (PCA) on our 30 MAG clusters and 135 genomes from marine isolates [4] based on log-transformed percentages of non-coding DNA, GC content, COG categories "transcription" (K), "signal transduction" (T), "defense mechanism" (V), "secondary metabolites biosynthesis" (Q) and "lipid transport and metabolism" (I). Only isolates belonging to the phyla (classes for proteobacteria) represented by MAGs were included. **b**, **c** Genome size versus percentage of non-coding DNA plotted for the same set of genomes (**b**), with a zoom-in on the smaller and denser genomes (**c**). **d**, **e** Number of sequence reads matching to the MAG clusters (in log scale) per 10,000 reads compared from each filter fraction in Dupont et al. [13]. **f** The ratio of matches between the 0.8–3.0 and the 0.1–0.8 μm fraction versus genome size, both in log scale

Oligotrophic bacterioplankton are characterized by streamlined genomes, i.e., small genomes with high coding densities and low numbers of paralogs [59]. For the few cultured oligotrophs, such as *Prochlorococcus* [60] and SAR11 [61], this coincides with small cell sizes. The small cells render high surface-to-volume ratios, beneficial for organisms that compete for very low concentration nutrients [62]. SAG sequencing has shown that genomic streamlining is a widely distributed feature among abundant bacterioplankton [3], contrasting with most cultured marine bacteria. Lauro et al. [2] identified genome features for predicting whether an organism or community is oligotrophic or copiotrophic. Ordination using some of these features (coding density, GC content and proportion of five COG categories [2, 3]) separated our MAG clusters from marine isolate genomes (Fig. 3a). The exceptions were isolates of picocyanobacteria, SAR11 and OM43 that overlapped with our MAG clusters, and the SAR92, OM182 and *Opitutaceae* MAG clusters that overlapped with the isolates. Hence, most of the MAGs displayed pronounced signs of streamlining. These features, with the exception of GC content, were found to be highly correlated with genome size (Figure S9 in Additional file 1), and genome size alone gave equally strong separation (Fig. 3b, c).

Interestingly, several of the *Bacteroidetes* MAG clusters appear to be streamlined, despite *Bacteroidetes* being generally described as copiotrophic [59]. One of them (BACL11), which represents a novel branch in the *Cryomorphaceae* (Fig. 1), has a particularly small genome (1.19 Mbp [range 1.16–1.21] MAG size, at 75 % estimated completeness) with only 4 % non-coding DNA. It encodes a smaller number of transporters than the other *Bacteroidetes* MAG clusters and only one type of glycoside hydrolase. It also has a comparatively low GC-content (33 %). However, the *Polaribacter* MAG cluster (BACL22), which has the largest genome and lowest gene density of the *Bacteroidetes* genome MAG clusters, has equally low GC content (32 %), as previously observed in planktonic and algae-attached *Polaribacter* isolates [63]. Since, in general, GC content correlates only weakly with both genome size and gene density (Figure S9 in Additional file 1), this may not be an optimal marker for genome streamlining. Supporting the impression that MAGs represent small and streamlined genomes, with little metabolic flexibility, most MAG clusters (25 of 30) encode rhodopsins (PF01036; Additional file 5), which allows them to adopt a photoheterotrophic lifestyle when their required substrates for chemoheterotrophy are not available.

By mapping shotgun reads from different filter fractions (0.1–0.8, 0.8–3.0 and >3.0 μm) from a previous spatial metagenomic survey of the Baltic Sea [13], we could investigate how MAG cluster cells were distributed across size fractions. Comparing counts of mapped reads between the 0.8–3.0 and 0.1–0.8 μm fractions showed that *Bacteroidetes* tended to be captured on the 0.8 μm filter to a higher extent than *Actinobacteria* (Fig. 3d). This bias could be driven by *Bacteroidetes* being, to a higher extent, attached to organic matter particles or phytoplankton. However, comparing the >3.0 μm with the 0.8–3.0 μm fraction showed a clear bias only for one of the *Bacteroidetes* clusters (BACL12; Fig. 3e). This cluster has the largest genomes (2.5 and 2.8 Mbp) of the reconstructed *Bacteroidetes* and is the only representative of the *Sphingobacteriales* (Fig. 1). *Sphingobacteria* have previously been suggested to bind to algal surfaces with the assistance of glycosyltransferases [64]. We did not find significantly more glycosyltransferases in BACL12 than in the other *Bacteroidetes*. Rather, it encodes a greater number of genes containing carbohydrate-binding module domains than the other clusters (x̄ = 12 in BACL12 versus 1.3 in the other *Bacteroidetes* and 1.4 in all clusters), which may facilitate adhesion to particles or phytoplankton [65].

Since only one *Bacteroidetes* MAG cluster was biased toward the >3 μm filter, attachment to organic particles doesn’t seem to be the main reason behind the difference in filter capture between *Bacteroidetes* and *Actinobacteria*, unless the particles are mainly in the 0.8–3.0 μm size range. Another possibility is that this bias reflects cell size distributions; each population has a specific size distribution that will influence what proportion of cells will pass through each membrane. Interestingly, the (0.8–3.0 μm)/(0.1–0.8 μm) read count ratio is correlated to genome size of the MAGs (Spearman rho = 0.76; *p* = 10^−5^; Fig. 3f), indicating a positive correlation between cell size and genome size.

The reason for the streamlining of genomes in oligotrophs is not known [59]. Lowered energetic costs for replication is one possibility. Despite the energetic requirements for DNA replication being low (<2 % of the total energy budget [66]), the extremely large effective population sizes of oligotrophic pelagic bacteria could explain selection for this trait [59]. Another possibility is spatial constraints. In *Pelagibacter* the genome occupies 30 % of the cell volume [61], so that cell size minimization may be constrained by the genome size. A strong correlation between cell and genome size for oligotrophic microbes would favor such an explanation. Further analyses with more reconstructed genomes and higher resolution of filter sizes could shed more light on the mechanisms behind genome streamlining.

### Seasonal dynamics

**Fig. 4:**
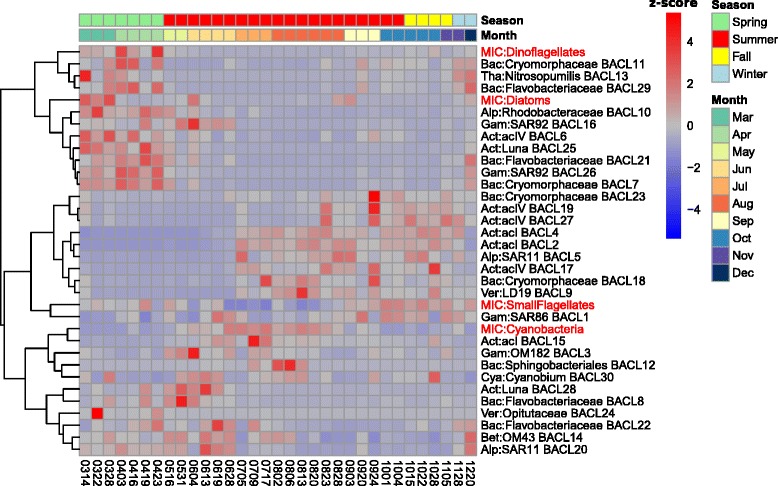
Seasonal dynamics of MAG clusters and phytoplankton. The heatmap shows the relative abundance (Z-score) of each MAG cluster in the time-series, based on calculated coverage from read mappings. In addition, the relative abundance of phytoplankton groups, assessed by microscopy, is shown for the same samples (prefixed by "*MIC*"). MAG clusters and eukaryotic groups were hierarchically clustered using Spearman correlations as shown in the dendrogram on the left. Colored rows at the top indicate month and season of each sample

Pronounced seasonal changes in environmental conditions with associated phytoplankton spring blooms are characteristic of temperate coastal waters. As is typical for the central Baltic Sea, in 2012 an early spring bloom of diatoms was followed by a dinoflagellate bloom, causing inorganic nitrogen to decrease rapidly; later in summer, diazotrophic filamentous cyanobacteria bloomed (Fig. 4; Figure S10 in Additional file 1). The only reconstructed picocyanobacteria genome (BACL30) peaked in early summer, between the spring and summer blooms of the larger phytoplankton. A similar pattern was previously observed for an operational taxonomic unit identical to the 16S rRNA gene of this reconstructed genome [39].

The seasonal dynamics of heterotrophic MAGs were highly influenced by the phytoplankton blooms, with different populations co-varying with different phytoplankton (Fig. 4). Phylum-level patterns were present, with a *Bacteroidetes*-dominated community in spring and early summer (7/9 *Bacteroidetes* MAG clusters), coinciding with the spring phytoplankton blooms, and *Actinobacteria* being more predominant in the second half of the year (8/9 *Actinobacteria*l MAG clusters). This pattern is in large agreement with observations by Lindh and colleagues in the same station in 2011 [41].

The pattern also broadly agrees with what is known for *Bacteroidetes*: being better adapted to feeding on complex carbohydrates abundant for the duration of phytoplankton blooms [44]. This was also reflected in the functional annotations, where *Bacteroidetes* MAGs contained several enzymes for degradation of polysaccharides and were enriched for certain aminopeptidases. For *Actinobacteria*, no such general correlation pattern to phytoplankton has been shown, but there are indications of association with and active uptake of photosynthates from cyanobacterial blooms [67, 68]. *Actinobacteria* MAGs, which were enriched in genes for the uptake and metabolism of monosaccharides such as galactose and xylose, became abundant as levels of dissolved organic carbon increased in the water (Fig. 4; Figure S10 in Additional file 1).

Besides these phylum- and order-level trends, temporal patterns were also observed at finer phylogenetic scales. The peaks of Luna clades coincide with spring phytoplankton blooms, while acI and acIV are more abundant in autumn, after these blooms. As previously reported for acI SAGs [6], cyanophycinase was found in two of the three acI MAG clusters, potentially allowing degradation of the storage compound cyanophycin synthesized by *Cyanobacteria*. These two acI MAG clusters (BACL2 and 4) became abundant in late July, as filamentous *Cyanobacteria*, which typically produce cyanophycin, started to peak in abundance (Fig. 4). In contrast, all acIV and Luna MAGs lacked this gene.

Furthermore, contrasting dynamics between members of the same clade, as exemplified by one acIV population blooming in spring, highlight that, despite the general similarities in their functional repertoire, lineage-specific adaptations allow different microniches to be occupied by different strains (Figs. 2 and 4). As an example, the spring blooming acIV BACL6 contained several genes for nucleotide degradation that were missing in the summer blooming acIV MAG clusters, such as adenine phosphoribosyltransferase, thymidine phosphorylase and pyrimidine utilization protein B. In addition, BACL6 contained genes *sulP* and *phnA* for uptake of sulfate and uptake and utilization of alkylphosphonate, respectively. These genes were also found in the spring blooming BACL25 (Luna clade), but were notably absent from the summer blooming acI, acIV and Luna MAG clusters. The capability to utilize nucleotides and phosphonates as carbon and phosphorous sources thus potentially set BACL6 and 25 apart from other closely related lineages.

The two SAR11 MAG clusters also showed contrasting seasonal patterns, with BACL20 being abundant in spring and peaking in early summer, while BACL5 appeared later and showed a stable profile from July onwards. Functional analysis showed that BACL5 contained several genes related to phosphate acquisition and storage that were missing from BACL20. These included the high-affinity *pstS* transporter, polyphosphate kinase and exopolyphosphatase, as well as the phosphate starvation-inducible gene *phoH*. BACL5 therefore appears better adapted to the low concentrations of phosphate found in mid- to late summer (Figure S10 in Additional file 1). In addition, proteorhodopsin was found in BACL5, but not in BACL20. However, since the latter consists of only one MAG, this gene may have been missed due to incomplete genome assembly.

### Biogeography of the brackish microbiome

**Fig. 5:**
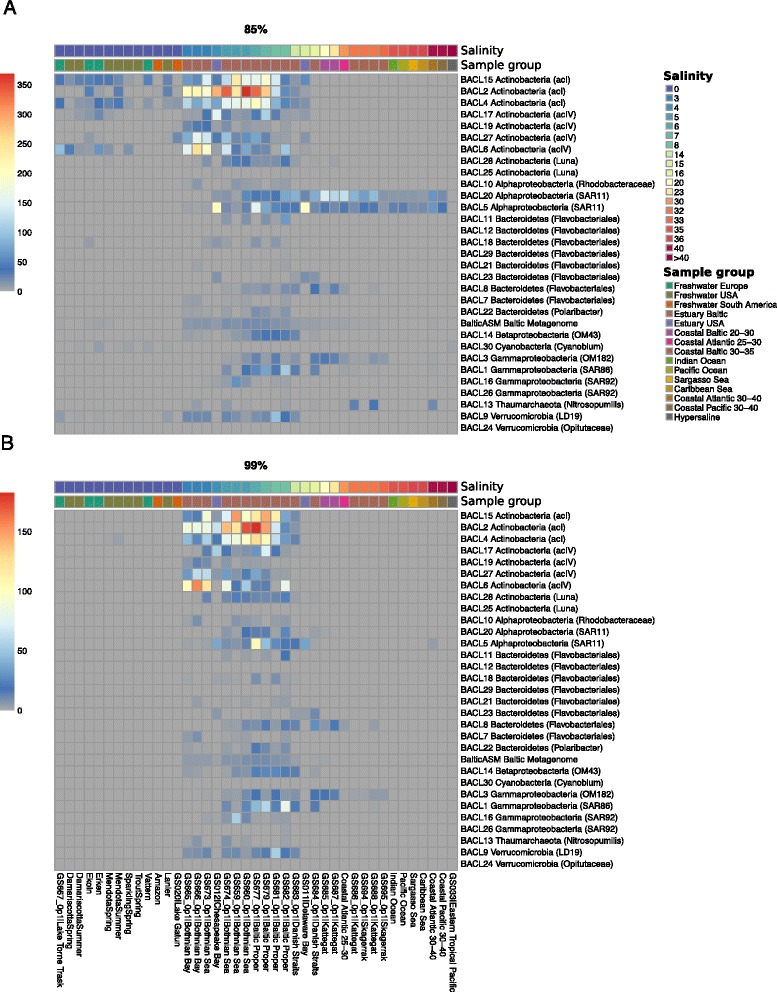
Biogeographical abundance profiles of MAGs. Heatmap plots showing the abundance of recruited reads from various samples and sample groups to each of the 30 MAG clusters as well as to a co-assembly of all samples in the time-series (“BalticAsm”) at the (**a**) 85 % and (**b**) 99 % identity cutoff levels. Shown values represent number of recruited reads/kb of genome per 10,000 queried reads. For clarity, several sample groups have been collapsed with recruitment values averaged over samples in the group. Sample groups are indicated by the lower color strip above the plot and samples are ordered by salinity (shown in the upper color strip). See Fig. S11 in Additional file 1 for full visualizations of samples

To assess how abundant the MAGs presented here are in other marine and freshwater environments around the globe, fragment recruitment was performed from a collection of samples comprising a wide range of salinity levels. At intermediate levels of sequence identity (85 %), different phylogenetic lineages recruit preferentially fresh or marine water fragments. Most markedly, SAR11, whose two MAG clusters belong to the marine subclade Ia (Figure S4c in Additional file 1), displays a clear marine profile, while acI and acIV *Actinobacteria* have a distinct freshwater signature (Fig. 5a; Figs. S11a and S12 in Additional file 1). In addition, MAGs belonging to *Bacteroidetes* and *Gammaproteobacteria* show signs of a marine rather than a freshwater signature that fits with the presence of the Na+-transporting NADH dehydrogenase in these lineages (Fig. S7 in Additional file 1). However, at a high identity level (99 %) only reads from brackish environments are recruited, including estuaries in North America (Chesapeake Bay, salinity = 3.5 practical salinity units (PSU); Delaware Bay, salinity = 15 PSU), to the exclusion of fresh and marine waters much closer geographically to the Baltic Sea (Fig. 5b; Figures. S11b and S12 in Additional file 1). Neither do Atlantic ocean waters sampled within a few days of these North-American estuaries show the same remarkable level of recruitment, indicating that salinity, not seasonality, is the determining factor in this pattern. Indeed, it is remarkable that BACL8 is placed phylogenetically as a single clade together with a SAG sampled in the brackish Chesapeake Bay (Figure S4b in Additional file 1). Despite being separated by thousands of kilometers of salt water, these cells share 99 % identity over the entire length of the SAG (70 % of MAG length), thus most likely representing the same species [42].

**Fig. 6:**
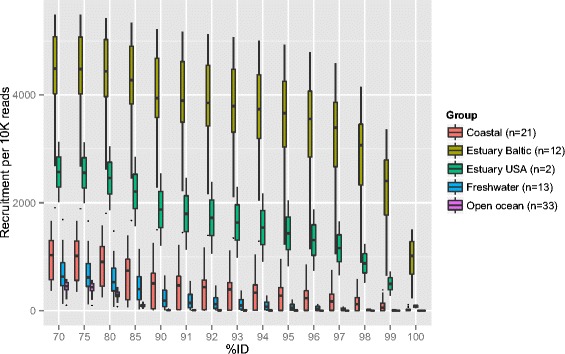
Fragment recruitment at different nucleotide identity, expressed as number of reads aligned per 10K reads. The reads from each sample were aligned to a co-assembly of all samples sequenced in this work, and recruitment values calculated at various percentage identity cutoffs. *Boxes* show average and variation of recruitment within sample groups. The number of samples included in each group is given in the legend

Some BACLs recruit markedly more fragments from the North American estuaries than others. This could be due to seasonal effects, since each North American station is represented by a single time-point, in which not all populations were equally abundant. The lower recruitment by certain BACLs could also reflect dispersal limitation and site-specific environmental differences, if these populations are never detectable in the North American estuaries. Nevertheless, our analysis shows that the reconstructed genomes recruited sequences primarily from brackish estuary environments at various levels of sequence identity, which holds true even when considering a co-assembly of reads from all the samples sequenced in this work. While most of the recruitment from brackish environments happens at 96–99 % identity, freshwater and marine environments don’t present significant recruitment until 80–90 % identity (Fig. 6).

The Baltic Sea is a young system, formed by the opening of the Danish straits to the North Sea in a long process between 13,000 and 8,000 years ago. The initially high salinity has slowly decreased due to the influx of freshwater from the surrounding area and the narrow connection to the open ocean, forming a stable brackish system around 4000 years ago, that now has a water renewal time of approximately 50 years [69]. Even considering fast rates of evolution for bacteria, the high degree of separation observed at the whole-genome level between the Baltic metagenome and global fresh and marine metagenomes cannot be explained by isolation in the Baltic alone. Based on the rates of evolution presented by [70], it would take over 100,000 years for free-living bacteria to accumulate 1 % genome divergence. These specialists must therefore have evolved before current stable bodies of brackish water, such as the Baltic Sea, the Black Sea and the Caspian Sea, were formed in the end of the last glacial period. Intriguingly, brackish-typical green sulfur bacteria have been observed in sediment layers of 217,000 years in the now highly saline Mediterranean [71], suggesting that brackish populations might migrate between these transient environments as salinity shifts. This is in agreement with the well documented separation between freshwater and marine species, which indicates that salinity level is a main barrier isolating populations (reviewed in [72]). Strains previously adapted to brackish environments and transported through winds, currents or migratory animals can thus proliferate and occupy available niches before fresh and marine strains can effectively adapt to the new environment.

The prokaryotic populations of the Baltic Sea thus appear to have adapted to its intermediate salinity levels via a different mode than its multicellular species, most of which are recently adapted to brackish environments from the surrounding fresh and marine waters [73, 74]. Indeed, while there is low multicellular species richness and intra-species diversity in the Baltic [75], suggestive of a recent evolutionary bottleneck, no such observation has been made for bacteria in the region [13, 40].

A key question that arises is what adaptations the populations have undergone that allowed the transition from freshwater or marine conditions to the brackish. When comparing aquatic metagenomes from different salinities at the whole community level, composition of functional genes is highly correlated with salinity (Figure S13 in Additional file 1), as has previously been observed [13, 76]. Brackish samples from the Baltic Sea cluster with North American estuary samples of similar salinities, surrounded by freshwater and marine samples at each end. Also, >50 % of the detected COGs correlate significantly in their abundance with salinity. The difference in gene composition between the brackish and the marine and freshwater metagenomes is, however, not necessarily reflecting genomic adaptations. Rather, it likely reflects that brackish communities comprise mixtures of lineages most closely related to freshwater and marine counterparts [40]. As more pairs of genomes of brackish and close freshwater or marine relatives become available, it will be possible to more directly assess how the populations have adapted to the altered salinity levels. Such analysis will potentially identify functional genes that have been independently gained or lost, or display elevated evolutionary rates, in multiple lineages during the transition from either freshwater or marine conditions to brackish.

## Conclusions

Here we present 83 genomes, corresponding to 30 clusters at >99 % nucleotide identity, reconstructed from metagenomic shotgun sequencing assemblies using an unsupervised binning approach. Many of the reconstructed genomes belong to lineages with no previous reference genome, including lineages known from 16S-amplicon studies to be highly abundant. We show that the seasonal dynamics of these bacterioplankton follow phylogenetic divisions, but with fine-grained lineage-specific adaptations. We confirm previous observations on the prevalence of genome streamlining in pelagic bacteria and our data indicate this is related to cell size minimization. Finally, we propose that brackish environments exert such strong selection for tolerance to intermediate salinity that lineages adapted to it flourish throughout the globe with limited influence from surrounding aquatic communities. The new genomes are now available to the wider research community to explore further questions in microbial ecology and biogeography.

## Materials and methods

### Sample collection, library preparation and sequencing

Water samples were collected on 37 occasions between March and December of 2012, at 2 m depth, at the LMO (N 56°55.851, E 17°03.640), 10 km off the coast of Öland (Sweden), using a Ruttner sampler. All samples are referred to in the text and figures by their sampling date, in the format yymmdd. Samples were filtered successively at 3.0 μm and 0.22 μm. The 0.22 μm fraction was used for DNA extraction. The procedures for DNA extraction, phytoplankton counts and chlorophyll a and nutrient concentration measurement are described in [41]. DNA (2–10 ng) from each sample were prepared with the Rubicon ThruPlex kit (Rubicon Genomics, Ann Arbor, Michigan, USA) according to the instructions of the manufacturer. Cleaning steps were performed with MyOne™ carboxylic acid-coated superparamagnetic beads (Invitrogen, Carlsbad, CA, USA). Finished libraries were sequenced in SciLifeLab/NGI (Solna, Sweden) on a HiSeq 2500 (Illumina Inc., San Diego, CA, USA). On average, 31.9 million paired-end reads of 2 × 100 bp were generated.

### Sequence data quality filtering and assembly

Reads were quality trimmed using sickle [77] to eliminate stretches where average quality scores fall below 30. Cutadapt [78] was used to eliminate adapter sequences from short fragments detected by FastQC [79]. Finally, FastUniq [80] was used to eliminate reads which were, on both forward and reverse strands, identical prefixes of longer reads (on average, 49 % of the reads from each sample). Each sample was then assembled separately, using Ray 2.1 (Ray Meta) [81] with kmer lengths of 21, 31, 41, 51, 61, 71 and 81. Contigs from each of these assemblies were cut up to 2000 bp in sliding windows every 100 bp using Metassemble [82], which keeps one copy of each subcontig, or two copies of subcontigs on the edges of contigs or of small (<1100 bp) contigs that are not cut, preventing loss of information due to low coverage. Subcontigs were then reassembled using 454 Life Science’s software Newbler (v.2.9; Roche, Basel, Switzerland), with default parameters (minimum overlap length of 40 bp, minimum overlap identity of 90 %). Assembly statistics for each MAG are available in Additional file 2.

Similar ensemble assembly approaches, where a de Bruijn assembler was used to repeatedly assemble reads using different kmer lengths, followed by overlap-layout consensus assembly of contigs from the individual assemblies, have been used and evaluated before on metagenome datasets and shown to generate longer contigs and higher accuracy than using only the de Bruijn assemblers [83–85]. Similarly to us, Luo et al. [83] used Newbler for the overlap-layout consensus step, although they used other de Bruijn graph assemblers than Ray. However, Ray has been evaluated for metagenome data with good results [81].

To assess the suitability of this approach to our particular dataset, an in silico spike-in experiment was performed by cutting up the genome of the *Pelagibacter ubique* isolate HTCC1062 in stretches of 1000 bp on a sliding window of 100 bp and adding the resulting artificial contigs to the background of contigs coming from Ray with all different k-mer lengths from sample 120322, one of the most deeply sequenced samples in this study. Newbler was run with default parameters. The Newbler report revealed that the sequence fragments from HTCC1062 were distributed over 86 contigs. Comparing these contigs with the reference genome using MUMmer [86] showed that 99.66 % of the 1,308,759 bp HTCC1062 genome was recovered. Only 0.2 % of bases in these 86 contigs did not map back to the reference genome. Over the whole alignment, 99.97 % of residues were identical between the reference genome and the assembled contigs. In contrast, in the absence of the spike-in, only 5.4 % of the HTCC1062 genome was covered by contigs with ≥90 % identity. The average identity of these alignments was 92.25 %.

### Binning of sequencing data and construction of MAGs

The quality-filtered reads of each sample were mapped against the contigs of all other samples using Bowtie2 [87], Samtools [88], Picard [89] and BEDTools [90]. Contigs from each sample were then binned based on their tetranucleotide composition and covariation across all samples using CONCOCT [36] and accepting contigs over 1000, 3000 or 5000 bp in length (three runs per sample). As in the original CONCOCT publication [36], contigs ≥20 kb in length were split into 10-kb fragments (sub-contigs) before CONCOCT was run. After binning, sub-contigs ending up in the same bin and that were adjacent in the original contigs were joined again. Prodigal [91] was used to predict proteins on contigs for each bin, and these were compared with the COG database with RPS-BLAST. The resulting hits were compared with a small set of 36 SCGs used by CONCOCT, only considering a protein hit if it covered more than half of the reference length. Bins were considered good if they presented at least 30 of the 36 SCGs, no more than two of which were in multiple copies. Another set of phylum-specific SCGs was used to evaluate each selected bin more carefully. Both the general prokaryotic SCGs and phylum-specific SCGs were selected such that they were present in at least 97 % of sequenced representatives within that taxon and had an average gene count of less than 1.03. For the phylum-specific SCGs, *Proteobacteria* was divided down to class level for increased sensitivity. The full list of SCGs used can be found in Additional files 2 and 3.

For each sample, only one CONCOCT run was chosen for downstream analysis. For most samples, the 1000-bp cutoff provided the maximum number of good bins, but samples 120705, 120828 and 121004 had best results with 3000 bp. This resulted in 83 good bins in total. As the same, or highly similar, genome could have been independently found in more than one sample, MUMmer [86] was used to compare all good bins against each other. The distance between two bins was set as one minus average nucleotide identity, given a minimum of 50 % bin coverage of the smallest bin in each pair. This procedure yielded 30 clearly distinct clusters (BACLs), independently of the clustering method used (average, full or single linkage).

### Abundance estimation and comparison of MAGs

The relative abundance of each MAG was estimated using the fraction of reads in each sample mapping to the respective MAG. Normalized on the size of that bin, this yielded the measure *fraction of reads per nucleotide in bin*. This measure was chosen since it is comparable across samples with varying sequencing output and different bin sizes. Using the CONCOCT input table, multiplying the average coverage per nucleotide with the length of the contig in question and summing over all contigs within a bin and within a sample gave the number of reads per bin within a sample. The fraction of reads in each sample mapping to each bin was then calculated by dividing this value with the total number of reads from each sample, after having removed duplicated reads.

### Functional analysis

Contigs in each genome cluster were annotated using PROKKA (v.1.7) [92], modified so that partial genes covering edges of contigs were included, to suit metagenomic datasets, and extended with additional annotations so that Pfam [93], TIGRFAMs (v.15.0) [94], COG [95] and Enzyme Commission [96] numbers were given for all sequences where applicable. The extended annotation was performed using homology search with RPS-BLAST. Metabolic pathways were predicted in MAGs using MinPath (v.1.2) [97] with the Metacyc database (v.18.1) [98] as a reference. Counts of COGs, Pfams, TIGRFAMs, enzymes or metabolic pathways were averaged within genome clusters (BACLs) and non-metric multidimensional scaling (NMDS) analysis was applied to the genome clusters based on either of these type of features, calculating pairwise cluster distances using Bray-Curtis dissimilarities. The NMDS analysis was performed using function ‘metaMDS’ in R package vegan (v.2.2-0) with the number of dimensions set to four after manual inspection of scree plots. Abundances of functional features were explored, and statistical analyses of functional differences between groups of MAGs performed using STAMP (v.2.0.9) [99] with multiple test correction using the Benjamini-Hochberg false discovery rate method.

### Taxonomic and phylogenetic annotation

Initial taxonomy assignment for each MAG was done with Phylosift [100]. Phylosift annotates contigs based on core genes and assigns a mass-probability to its classification. To go from contig-level annotation to MAG-level annotation, this mass-probability was weighted by the number of bases in each contig. The last common ancestor for all annotations reaching at least 30 % of weighted support was considered as bin-level annotation. This provided 86–100 % support to phylum-level classification and 66–100 % at the class level, except for the three bins in BACL9, which had >40 % support for classification as virus. To improve the resolution of annotations, classification of 16S rRNA genes was also used. Complete or partial 16S genes were identified on contigs using WebMGA [101]. Further, since rRNA is difficult both to assemble and to bin, a complementary approach was used where partial 16S rRNA genes were assembled for each MAG using reads classified as SSU rRNA by SortMeRNA [102], but whose paired-end read was assembled in another contig belonging to the same MAG. The identified and reconstructed 16S fragments were classified with stand-alone SINA 1.2.13 [103] and by Blasting against the data by Newton et al. [55].

Using the information provided by Phylosift and 16S analysis, relevant isolate genomes and SAGs were selected. These were combined with all complete prokaryotic genomes in the RefSeq database. Prodigal was used for protein prediction in each genome. These proteomes, together with the proteomes of our MAGs, were used for phylogenetic tree reconstruction using Phylophlan [43]. Phylophlan’s reference database was not used as we noticed that, in instances where genomes that were already present in the reference were processed by us and added, they tended to branch closer to the MAG than otherwise, thus indicating a role of protein prediction method in the phylogeny. The tree visualizations displayed here were generated with Archaeopteryx [104] and FigTree [105]. For the sake of clarity, not all species included in the tree are maintained in the overview or clade-specific insets. Since the distance between MAGs and their nearest neighbors in database: RefSeq were, as a rule, too large for average nucleotide identity (ANI) calculation, we adopted Genome BLAST Distance for this comparison, using the online Genome-to-Genome Distance Calculator [106].

### Genome streamlining analysis

The dataset of marine microbial isolates from [4] was downloaded from CAMERA [107]. These were functionally annotated in the same way as the MAGs. For streamlining analysis, the GC content, genome length, and average fraction of non-coding nucleotides were calculated. To avoid bias of shorter contigs, the average fraction of non-coding nucleotides was only based on sequences longer than 5000 nucleotides. For clarity, only genomes belonging to the same phyla as our reconstructed MAGs were included in the analysis. For quantifying how MAG cluster cells were distributed across filter size fractions in [13], 10,000 random reads were sampled from each size fraction from 21 samples and aligned to the MAGs by BLAST, using 95 % identity and alignment length of 100 bp as cutoff.

### Fragment recruitment

Fragment recruitment [12] was used to estimate the presence of the reconstructed MAGs in various locations around the globe. We selected a total of 86 metagenomic samples obtained from a wide range of salinity levels and geographic locations (Table 2). The missing salinity value for Delaware Bay (GS011) was set to 15 PSU after consulting the Delaware Bay Operational Forecast System [108]. All samples were sub-sampled to 10,000 sequences, each 350 bp in length, and all reads were queried against a database of the reconstructed genome bins using Blast + (v.2.2.30). Non-coding intergenic sequences were excluded by using only the nucleotide sequences of predicted open reading frames. Only samples comprising the 0.1–0.8 μm filter fraction were used and only hits with e-value < 1e-5 and alignment length >200 bp were considered. For visualizations, the number of hits for MAGs in each sample was normalized against the total size (in base pairs) of the MAG. These normalized counts were then averaged over the MAGs of each BACL.

**Table 2:** Metagenomic projects used as queries for biogeographic fragment recruitments

Project	Project ID	Samples	Salinity range (PSU)	Reference
Global Ocean Sampling Expedition	CAM_PROJ_GOS	56	0.1–37, 63 (hypersaline)	[12]
Global Ocean Sampling Baltic Sea	CAM_P_0001109	19	0–34	[13]
Freshwater metagenomes	PRJEB4844	9	0	[109]
Lake Lanier metagenome by 454	SRR063691	1	0	[76]
Metagenomics of the Amazon	SRR091234	1	0	[113]

### Functional gene content analysis in metagenomes

Metagenomic assemblies of the Global Ocean Sampling expedition (http://data.imicrobe.us/project/view/26) [12], the Global Ocean Sampling Baltic Sea (http://data.imicrobe.us/project/view/114) [13], and of nine metagenomic samples from freshwater lakes in Sweden and USA (Sequence Read Archive study ERP004168) [109] were concatenated. All three assemblies were constructed using Newbler (454 Life Science, Roche, Basel, Switzerland) with default settings. A total of 24,041,069 genes were identified in the concatenated assembly using Prodigal [110] with default settings, and were given COG annotations by RPSBlast against the CDD database [111]. Samples used for the assemblies were sub-sampled to 100,000 sequences and samples with fewer sequences were excluded, resulting in a total of 114 samples. In addition, one sample per month (ten in total) was chosen from the LMO time-series (this study). To make the 454 and Illumina datasets comparable, all sequences were cut to 90 nucleotides. Genes were quantified by blasting (Blastn) the 100,000 sub-sampled reads from each sample against the concatenated assembly. Best hits were counted if the alignment had >90 % identity over an alignment of >63 bp. Finally, counts were summed per COG annotation for each sample. For the principal coordinates analysis, pairwise sample distances were calculated using Spearman correlations of COG counts.

### Availability of supporting data

The metagenome sequencing reads have been submitted to NCBI’s Sequence Read Archive under accession numbers SRR2053273–SRR2053308. Contigs for each MAG are available at NCBI’s Whole Genome Shotgun database under accession numbers LIAK00000000–LIDO00000000.

### Ethics approval

Ethics approval was not required for the study.

## Additional files

Additional file 1:
**Figure S1.** The assembly and binning workflow. **Figure S2.** The correlation between various assembly parameter qualities and number of MAG generated. **Figure S3.** Heatmap of the similarity between each of the 83 MAGs generated. **Figure S4.** Phylum and class-level insets of the phylogeny of each MAG. **Figure S5.** NMDS of BACL based on their functional gene content. **Figure S6.** Heatmap of the abundance of glycoside hydrolases in each BACL. **Figure S7.** Heatmap of the abundance of transporters in each BACL. **Figure S8.** Core genome and pigment phylogeny of picocyanobacteria including BACL30. **Figure S9.** Pairwise scatterplots of various genomic features in MAGs and isolates. **Figure S10.** Line plots of physico-chemical parameters of the studied station throughout the year. **Figure S11.** Heatmap of the fragment recruitment by each BACL of various aquatic metagenomes from previous studies. **Figure S12.** Fragment recruitment dot plots for selected MAGs. **Fig. S13** Principal coordinates analysis of functional gene content of various metagenomes showing salinity explains most of the variance. **Table S1.** ANOSIM results of clustering MAGs according to their coding potential. (PDF 6175 kb) Additional file 2:
**Summary statistics of MAGs.** For each MAG, the number of bases, number of contigs, N50, N50 length, N90, N90 length, length of longest contig, coverage, taxonomy and copy number of single-copy core and phylum-specific COGs are reported. Universal single-copy genes are defined here as present in >97 % of relevant genomes with an average copy number <1.03. The name of each MAG has the format yymmdd-bin##, where yymmdd is the date of sample collection and ## is a unique numerical identifier assigned by CONCOCT. (XLSX 99 kb) Additional file 3:
**Depicting COGs used as phylum-specific, or class-specific for proteobacteria, single-copy genes (SCGs).** A COG is considered a SCG for a clade if it is present in >97 % of the respective reference genomes with an average copy number <1.03. A COG marked with a 1 is considered an SCG for the clade corresponding to that column. Only clades with at least three genera represented by reference genomes are included in the table. (XLS 320 kb) Additional file 4:
**Phylogenetic tree in Newick format including all complete prokaryotic genomes in NCBI [** 112 **], all approved reconstructed bins (MAGs), and selected single amplified genomes, metagenome bins and isolate genomes from previous studies.** (NWK 191 kb) Additional file 5:
**Counts of COGs, PFAMs, TIGRFAMs and enzymes in each MAG.** (XLSX 2595 kb) Additional file 6:
**Mean counts of transporter genes encoded in each MAG cluster (BACL).** (XLSX 97 kb)

## Abbreviations

BACL: Baltic Sea genome cluster
bp: base pair
COG: Cluster of Orthologous Groups
LMO: Linnaeus Microbial Observatory
MAG: metagenome-assembled genome
NMDS: non-metric multidimensional scaling
PSU: practical salinity unit
SAG: single-amplified genome
SCG: single-copy gene
