# Supplementary material for: Metagenome-assembled genomes uncover a global brackish microbiome
Source: Genome Biol. 2015 Dec 14;16:279. doi: 10.1186/s13059-015-0834-7 (PMC4699468; doi:10.1186/s13059-015-0834-7)
Supplement: Additional file 1: — Figure S1. The assembly and binning workflow. Figure S2. The correlation between various assembly parameter qualities and number of MAG generated. Figure S3. Heatmap of the similarity between each of the 83 MAGs generated. Figure S4. Phylum and class-level insets of the phylogeny of each MAG. Figure S5. NMDS of BACL based on their functional gene content. Figure S6. Heatmap of the abundance of glycoside hydrolases in each BACL. Figure S7. Heatmap of the abundance of transporters in each BACL. Figure S8. Core genome and pigment phylogeny of picocyanobacteria including BACL30. Figure S9. Pairwise scatterplots of various genomic features in MAGs and isolates. Figure S10. Line plots of physico-chemical parameters of the studied station throughout the year. Figure S11. Heatmap of the fragment recruitment by each BACL of various aquatic metagenomes from previous studies. Figure S12. Fragment recruitment dot plots for selected MAGs. Fig. S13 Principal coordinates analysis of functional gene content of various metagenomes showing salinity explains most of the variance. Table S1. ANOSIM results of clustering MAGs according to their coding potential. (PDF 6175 kb) [file 13059_2015_834_MOESM1_ESM.pdf]

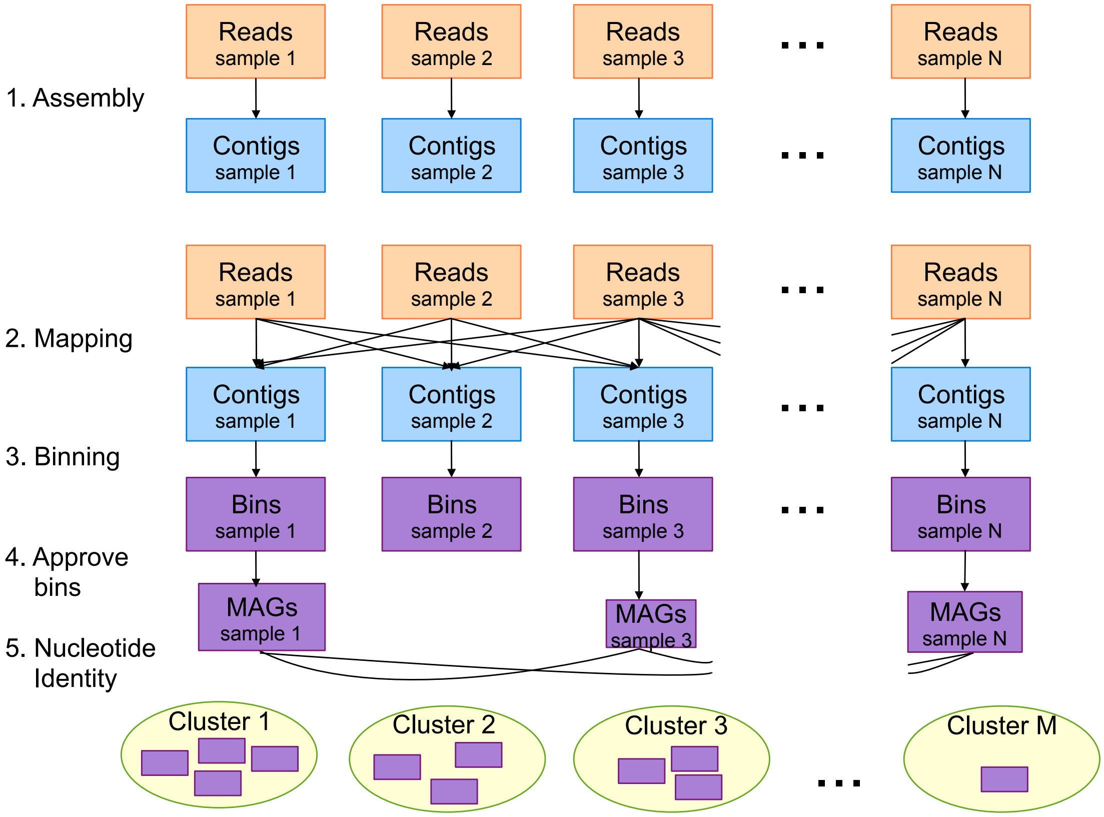

Figure S1. Overview of the genome reconstruction procedure. 1) Reads from each sample are assembled separately. 2) Reads from each of the samples are mapped onto the contigs of all samples, generating a coverage profile for each contig. 3) Together with tetranucleotide composition, these profiles are used to bin contigs in each sample with CONCOCT. 4) Completeness and purity of the bins are evaluated using single-copy housekeeping genes, and approved bins are selected. 5) Approved bins (metagenome-assembled genomes; MAGs) from all samples are cross-compared using sequence identity and highly similar (>99% identity) MAGs are clustered.

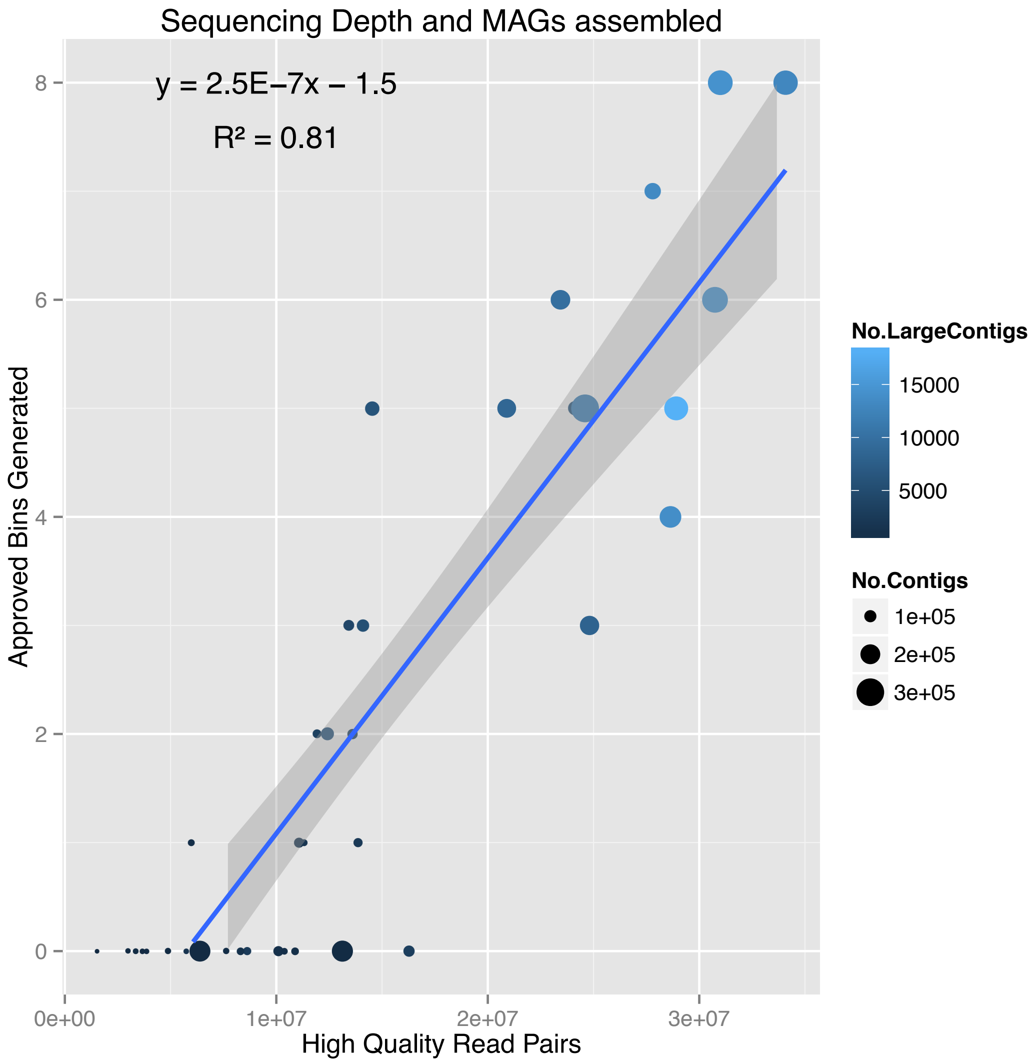

Figure S2. The number of approved bins generated from each sample is directly proportional to the number of sequencing reads passing quality control, the number of contigs assembled and the number of contigs of at least 1000 bp (LargeContigs).

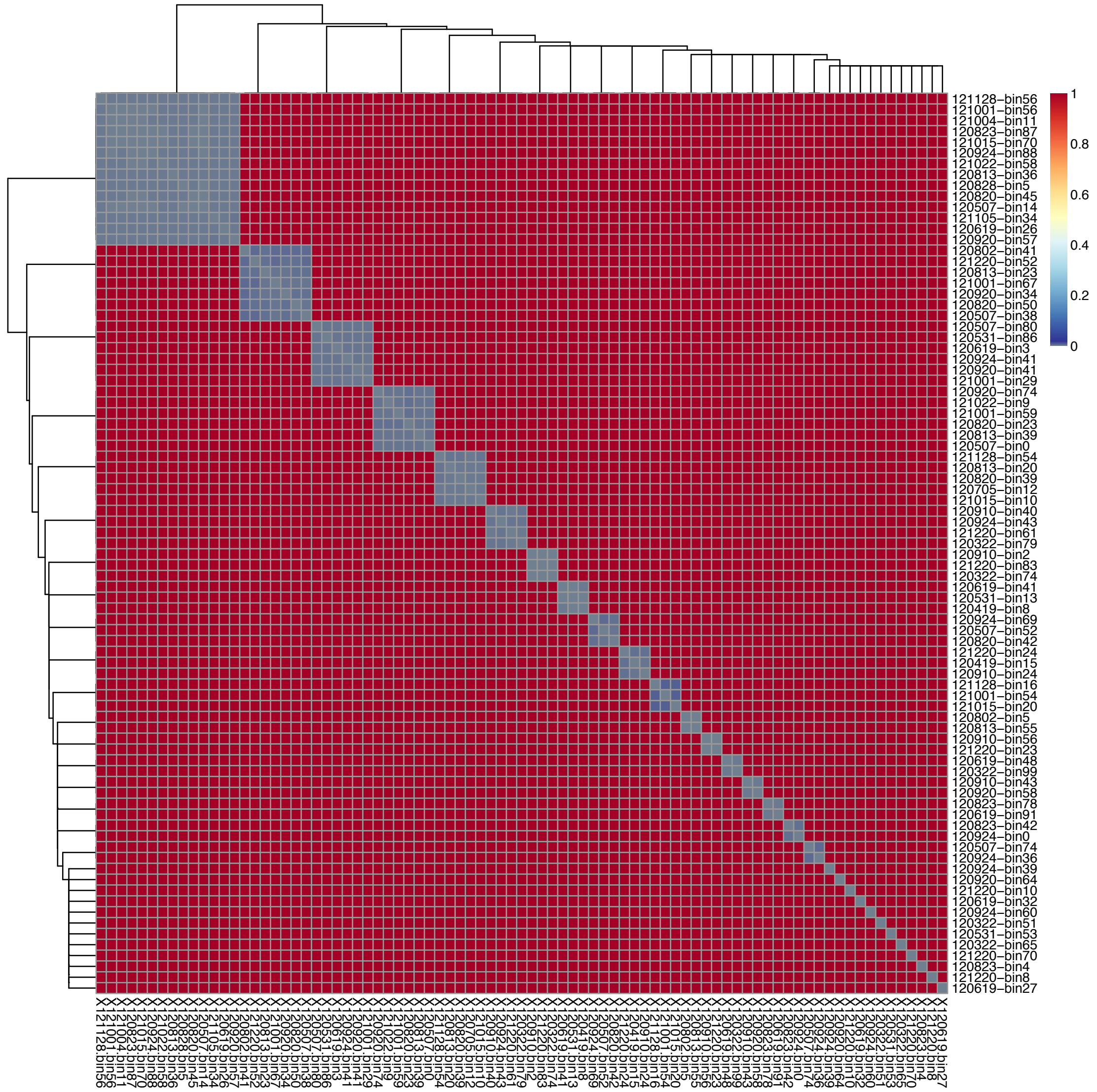

Figure S3. Average-linkage clustering of MAGs. Distance is defined as 1 minus the average nucleotide identity between MAGs, or set to 1 when the coverage of the larger MAG over the smaller is less than 50%.

Figure S4. Insets of the global phylogenetic tree, detailing the placement of the MAGs generated in different phyla/classes. Shimodaira-Hasegawa support for each node partition is displayed. The full tree from which these subtrees were extracted is available for download in newick format as Additional File 4.

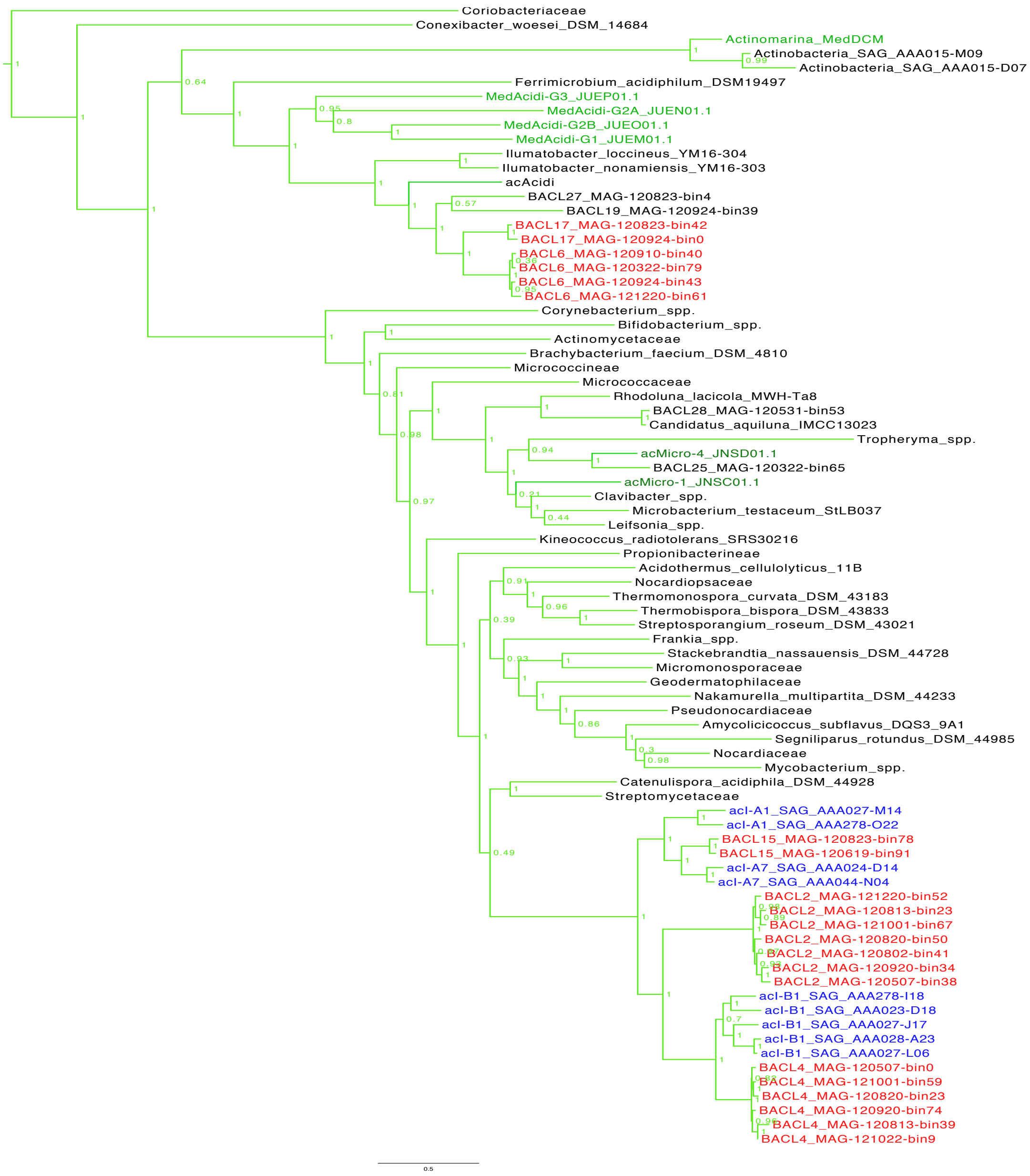

(**a**) *Actinobacteria*. Reconstructed actinobacterial genomes belong to the lineages acI, acIV and Luna. Ac-I and Luna belong to the order *Actinomycetales*, while ac-IV belong to *Acidimicrobiales*. Isolates of the Luna clade have had their genome sequenced [114, 115]. Isolation of acI and acIV has been unsuccessful, but SAGs have recently been published for acI-A and acI-B [6, 116]. Reconstructed 16S fragments placed MAGs in lineages acI-A, acI-B, acI-C, acIV and Luna [55, 117–120]. Consistent with previous estimates based on a few contigs identified as acIV [121] these MAGs are relatively low GC (average 53%).

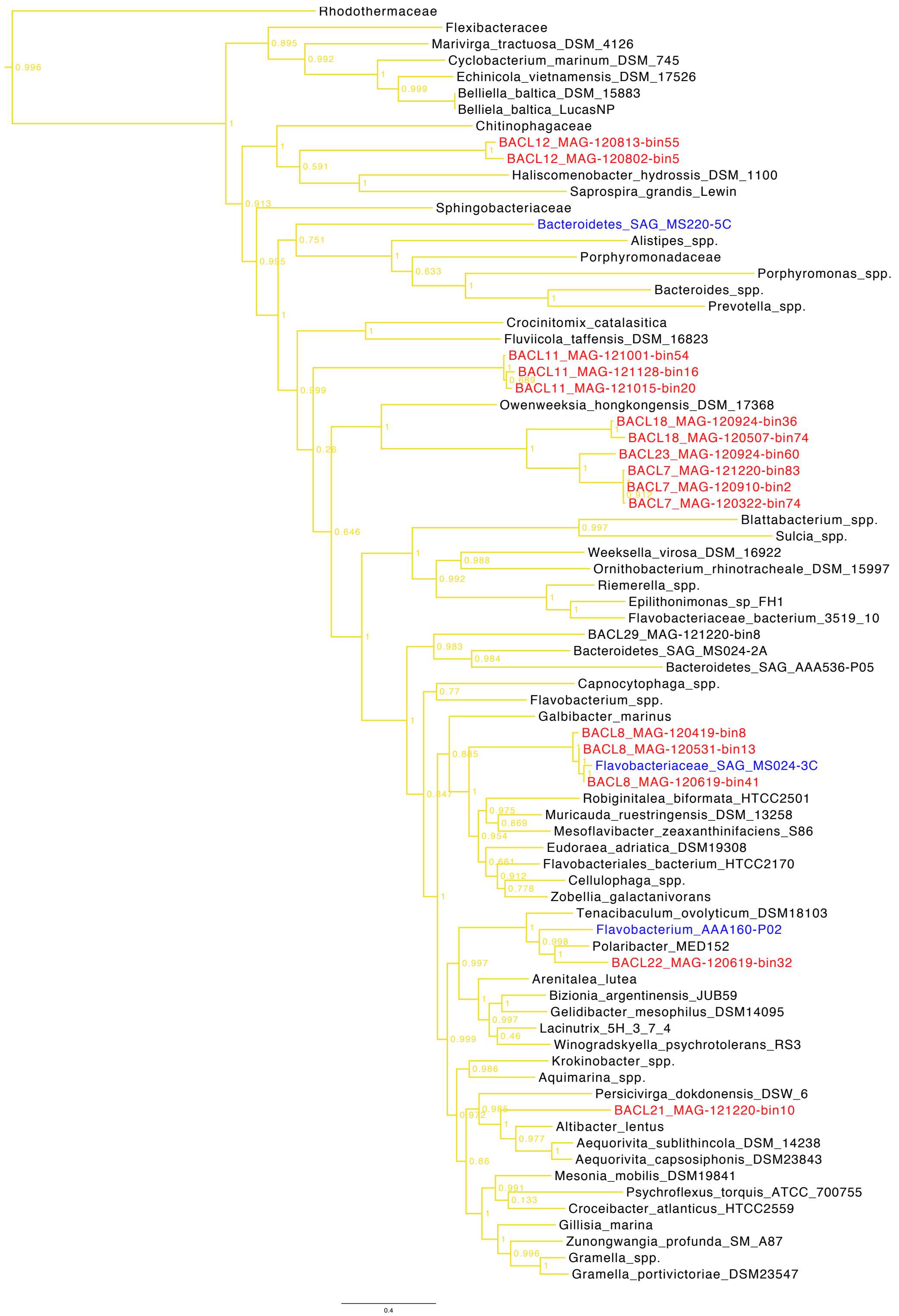

(**b**) *Bacteroidetes*. The reconstructed *Bacteroidetes* genomes belong to the families *Flavobacteriaceae* and *Cryomorphaceae,* and to the order *Sphingobacteriales*. *Cryomorphaceae* is a poorly studied family with just three isolate genomes and one SAG available [122], not closely related to the clusters that form three major branches presented here.

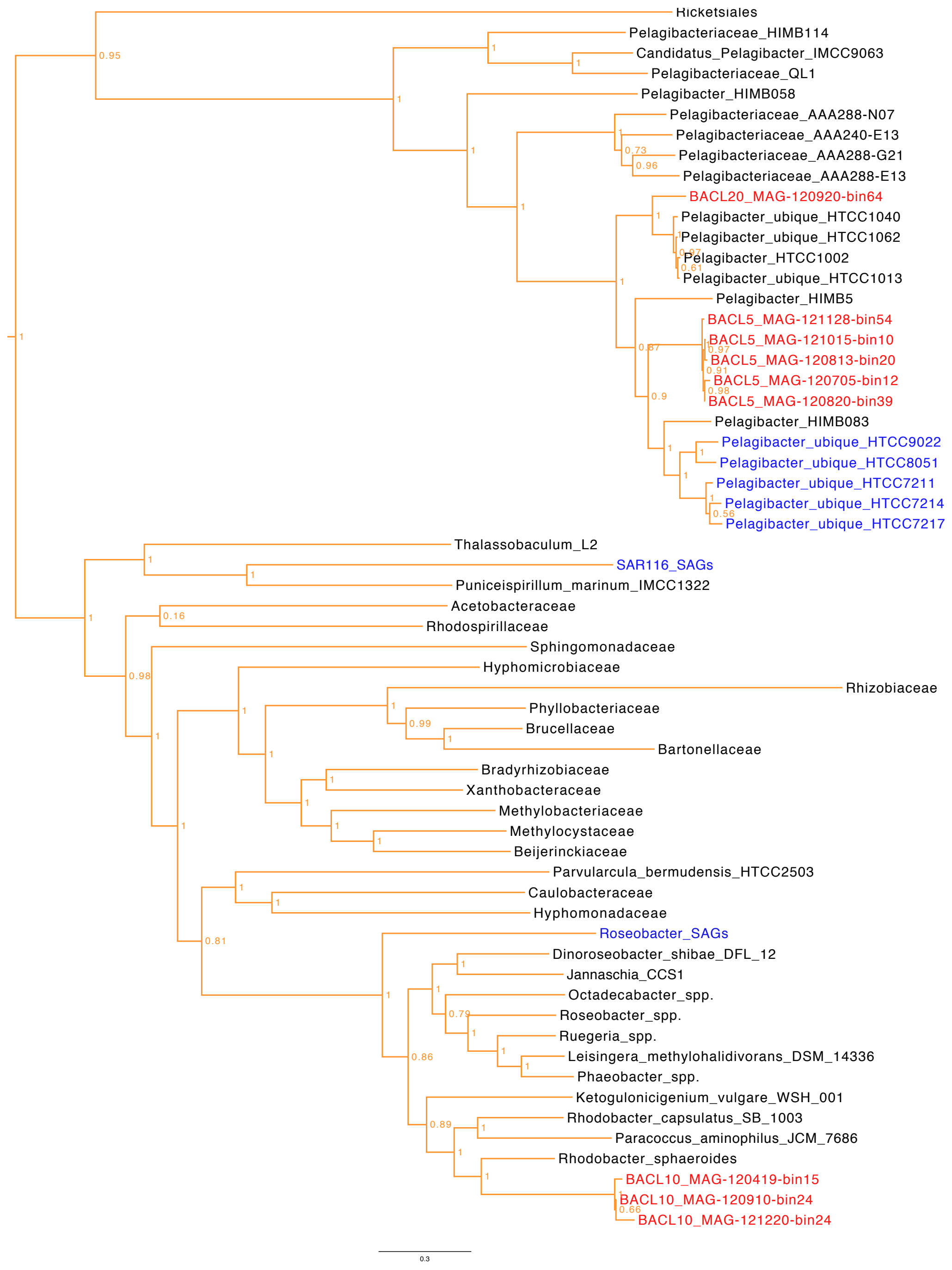

(**c**) *Alphaproteobacteria*. SAR11 and *Rhodobacteraceae* clusters were constructed. The two *Pelagibacteriaceae* (SAR11) clusters are clearly placed in the marine Ia clade. They are likely representatives of a brackish/marine subclade previously suggested to belong to clade ‘Ib’ (see Figure 4A in [13]), but are shown here to be part of Ia.

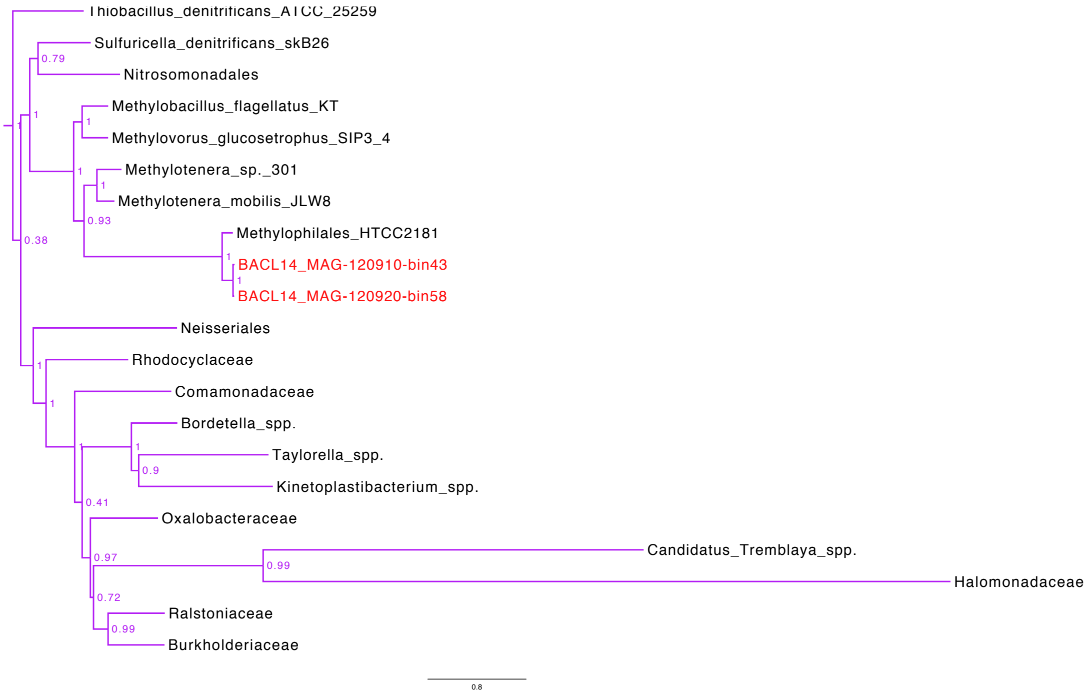

(**d**) *Betaproteobacteria*. One OM43 MAG was constructed (BACL14). Reference genomes have been available for this methylotrophic clade, from the Pacific Ocean and lake sediments [123–125]. BACL14 encoded a methanol dehydrogenase gene and genes for formaldehyde assimilation, and was sparse in uptake system genes, as expected for the simple obligate methylotrophs with small genomes of clade OM43 [123].

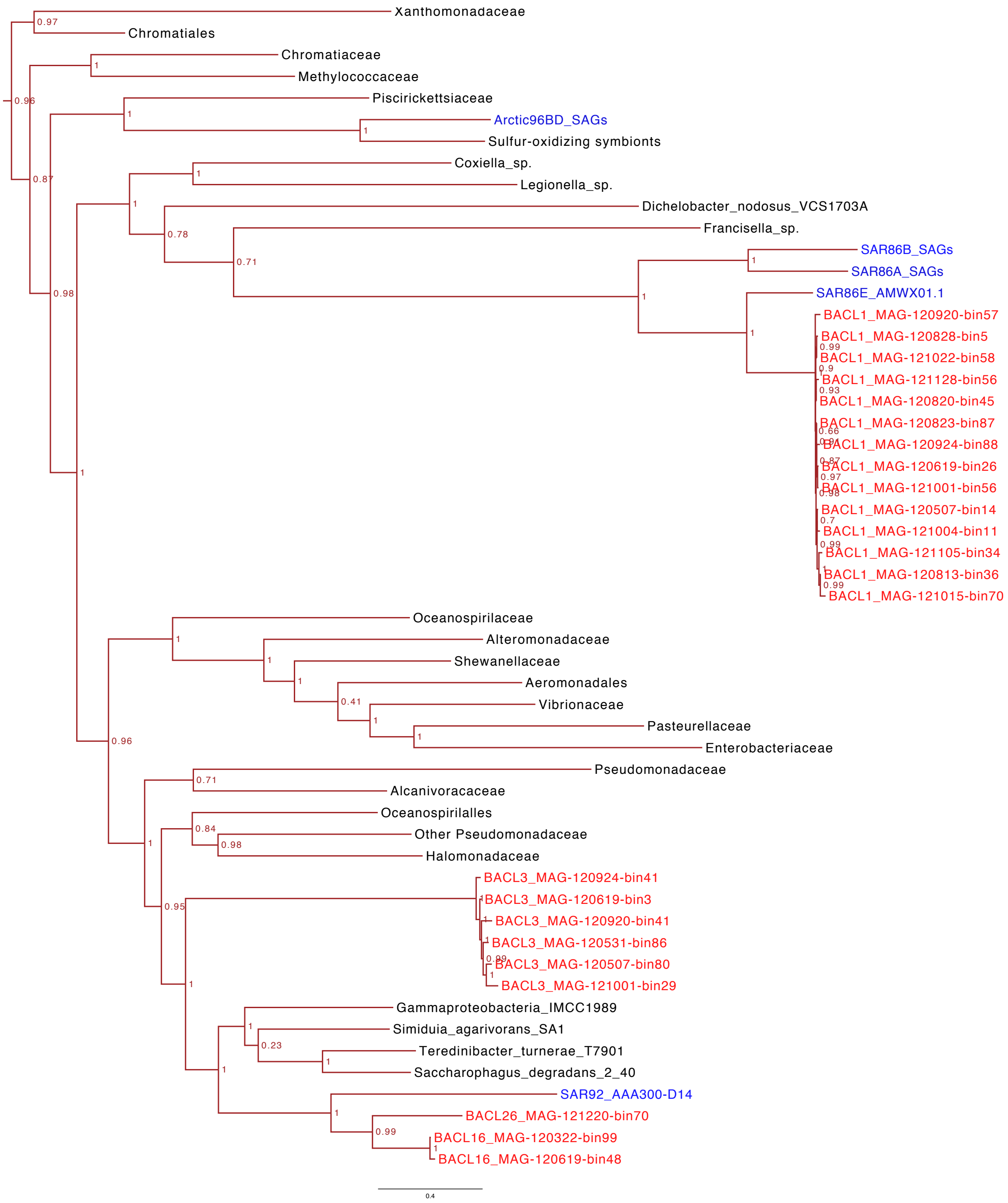

(**e**) *Gammaproteobacteria*. Known marine clades of *Gammaproteobacteria* were reconstructed, including SAR86, SAR92, and OM182. No reference genome was previously available for the latter.

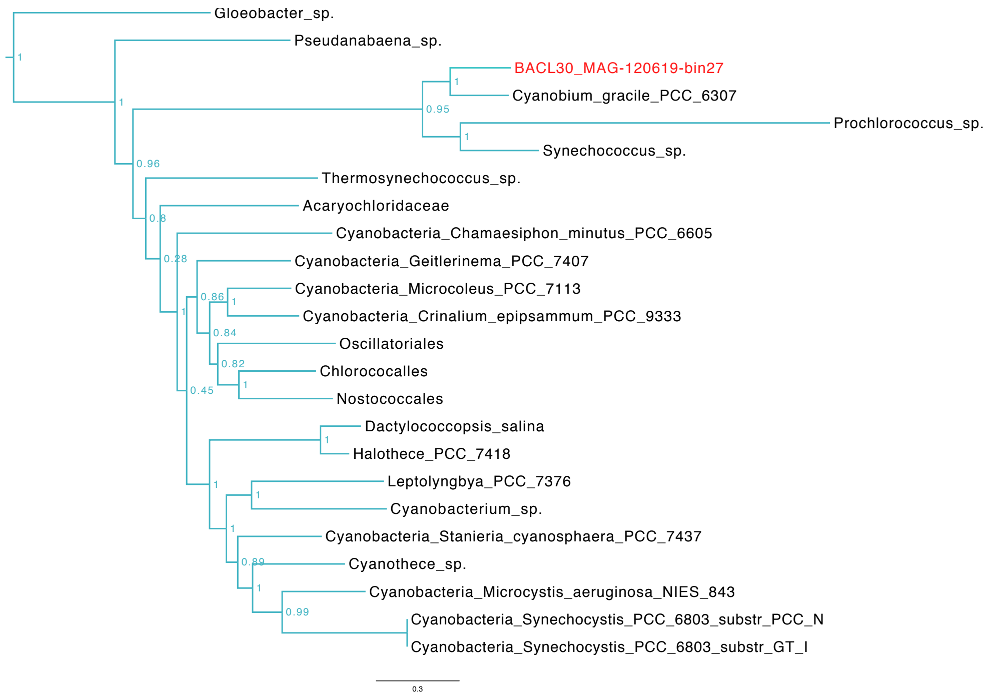

(**f**) *Cyanobacteria*. The *Cyanobacteria* genome reconstructed here belongs to a picocyanobacteria with 100% 16S identity to various freshwater *Synechococcus/Cyanobium* [126]. An operational taxonomic unit of identical 16S has also been observed to be abundant across the Baltic with a strong spatial correlation with the previously described Verrucomicrobia MAG “Candidatus Spartobacteria baltica” [27].

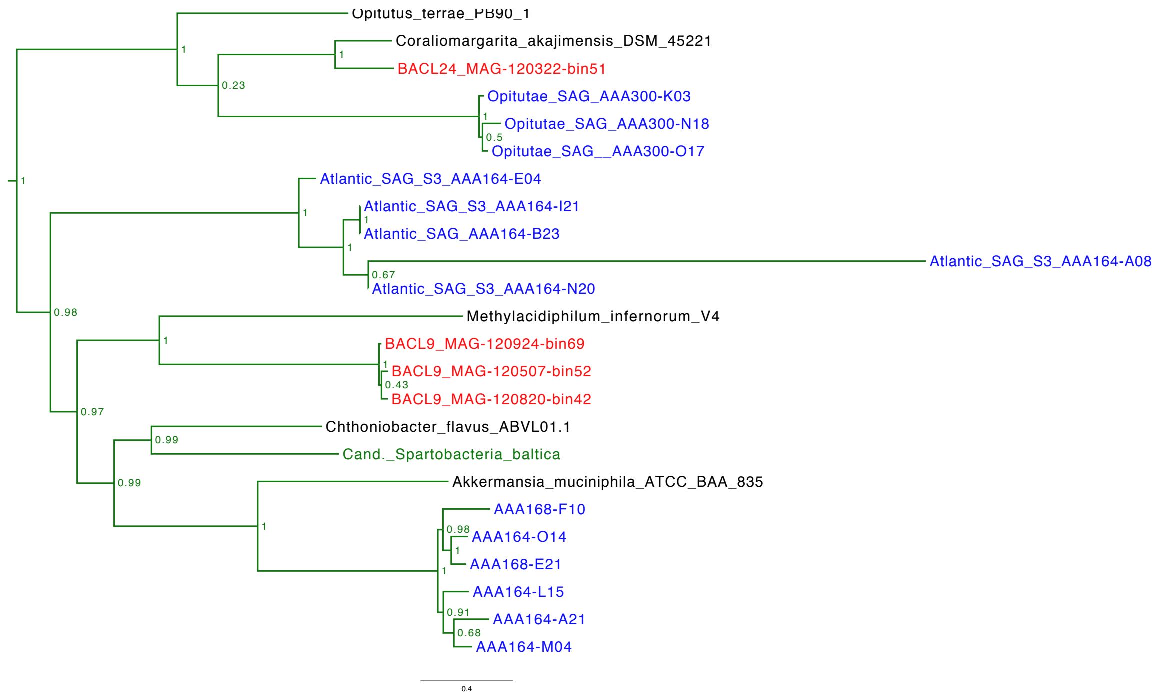

(**g**) *Verrucomicrobia*. The *Verrucomicrobia* phylum has been divided into five monophyletic subdivisions [127], but shortly afterwards a novel freshwater subdivision (LD19; [128]) and a subdivision consisting of acidophilic methanotrophs [129] were found. BACL24 is phylogenetically placed within the family *Opitutaceae* (Subdivision 4), closest to the freshwater isolate *Coraliomargarita akajimensis* [130]. BACL9 forms a sister branch to *Methylacidiphilum infernorum*, but their 16S rRNA genes are only 84% identical. Instead, 16S positions BACL9 as a member of LD19.

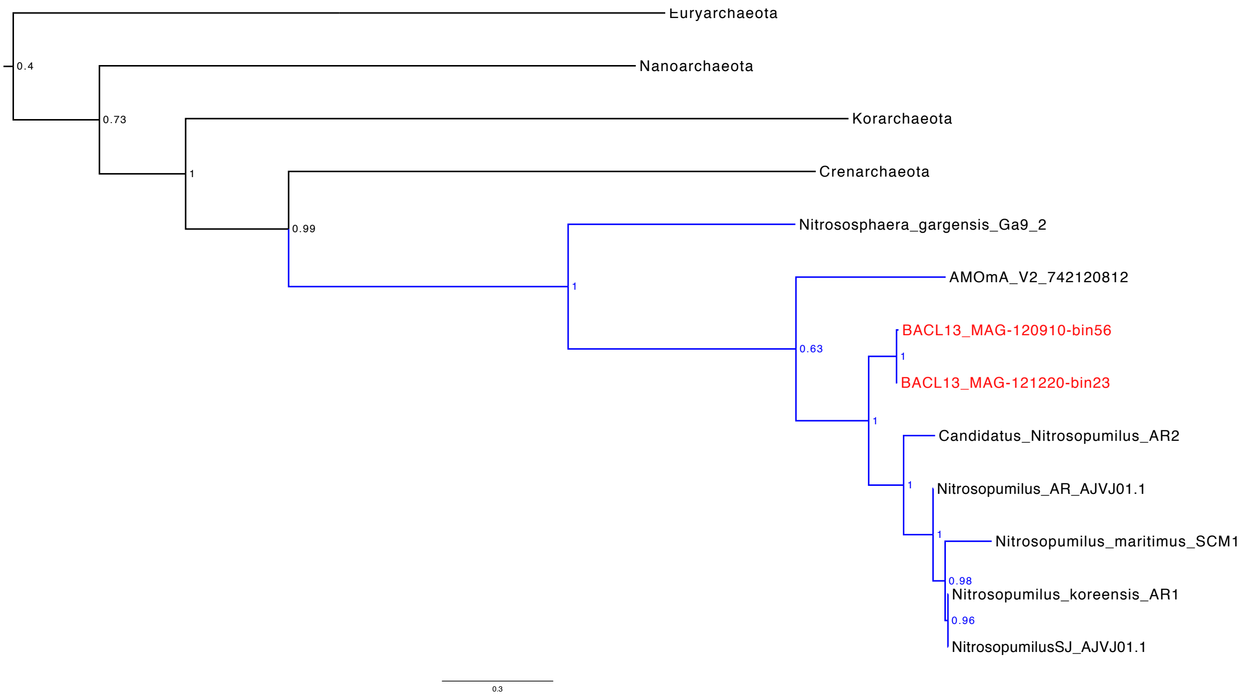

(**h**) *Thaumarchaeota*. *Thaumarchaeota* are very abundant in the global ocean [131], where they play important roles in the nitrogen and carbon cycles by driving ammonia oxidation [132]. A single archaeal genome cluster was reconstructed, whose 16S rRNA gene is 99% identical to marine lineages of *Nitrosopumilus maritimus* [133, 134]). *N. maritimus* are known to be abundant in Baltic Sea suboxic waters and this MAG has 98% identity to the previously described Baltic *N. maritimus* GD2 (98%; [135]). However, a whole genome comparison does not support the placement of these MAGs in the same species as *N. maritimus*, but rather in the same genus or family. Like all *Nitrosopumilus,* BACL13 encodes the amoA ammonia monooxygenase gene. In addition, it encodes urease genes, indicating the capability to utilise urea for nitrification, as previously observed in Arctic *Nitrosopumilus* [136].

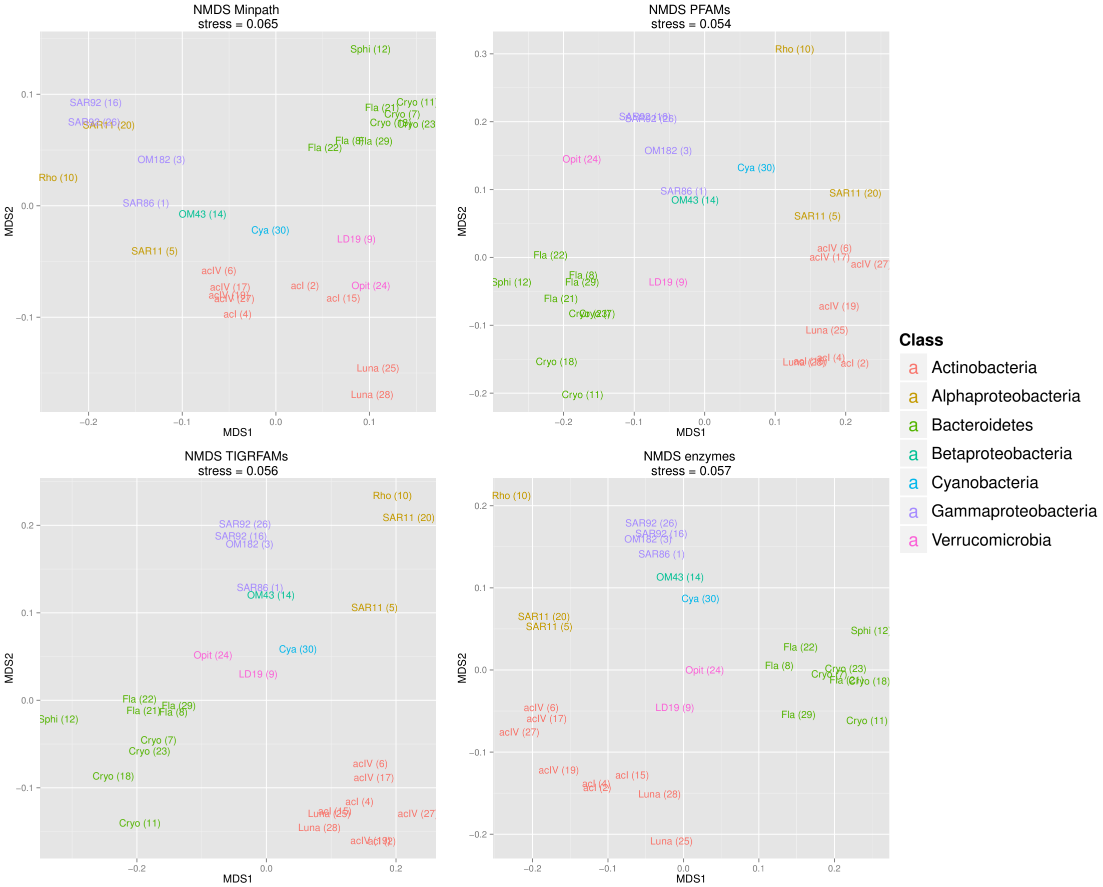

Figure S5. Non-metric multidimensional scaling plots based on functional annotations based on enzymes (“enzymes”), metabolic pathways (“Minpath”), and protein families (“PFAMs” and “TIGRFAMs”). Genome clusters are displayed with abbreviated lineage names and cluster numbers in parentheses and further colored according to Phyla/Class.

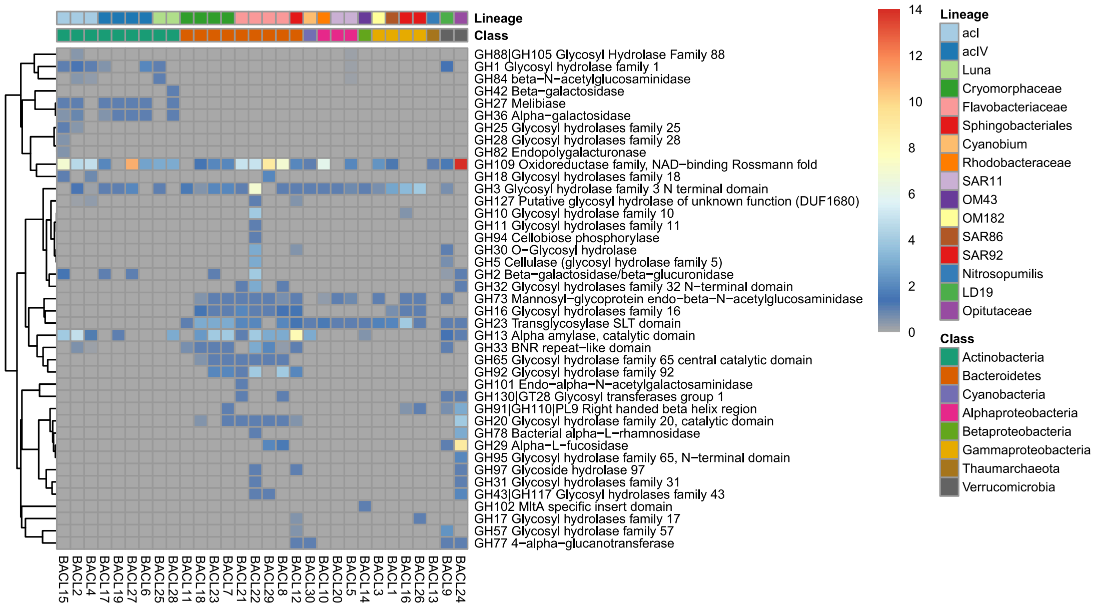

Figure S6. Abundance of glycoside hydrolase genes in MAG clusters. Counts were averaged over MAGs in each genome cluster.

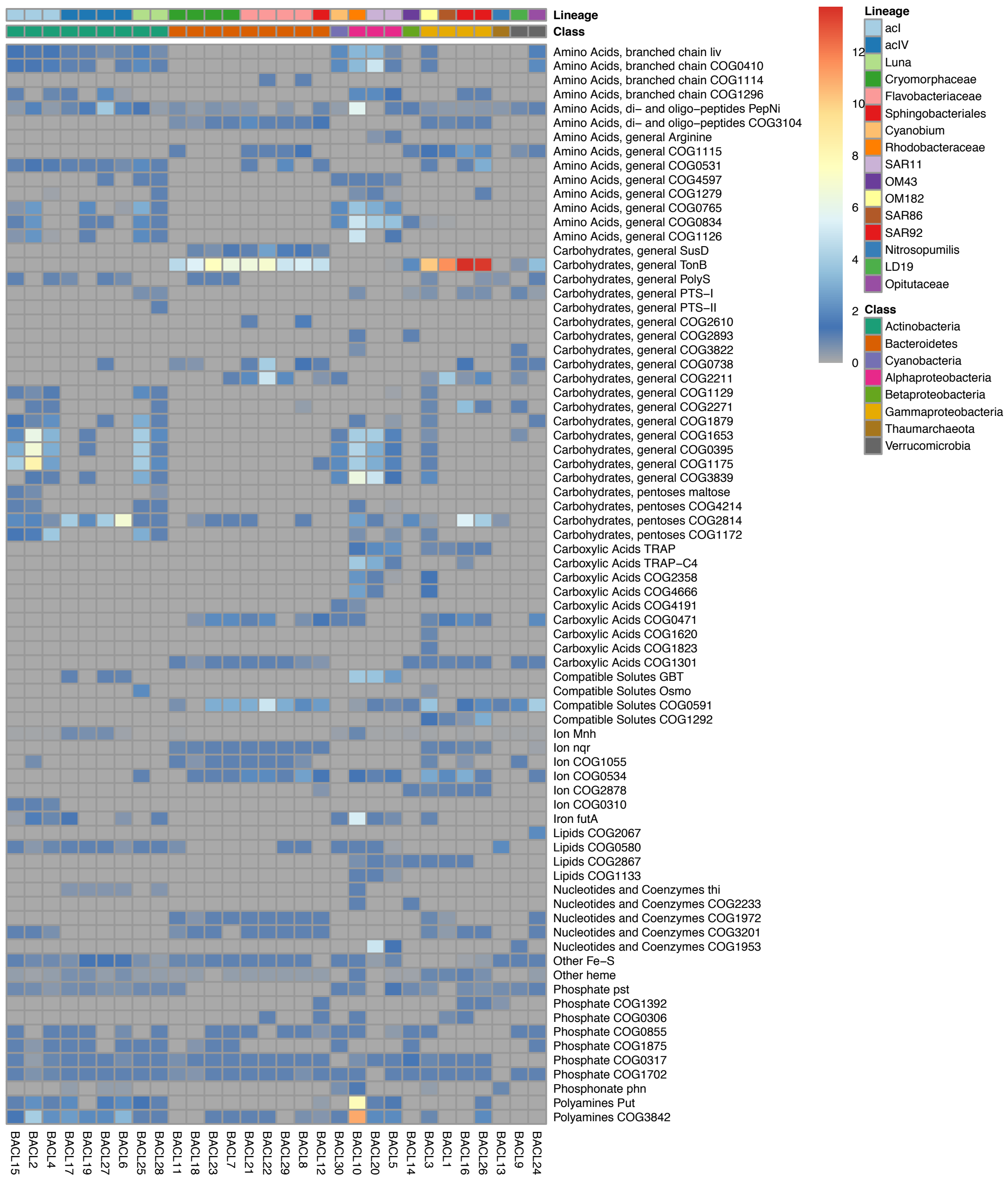

Figure S7. Abundance of transporters in MAG clusters. Counts of transporter genes were averaged over MAGs in each genome cluster and over transporter types where applicable (see also Additional file 6). Important general patterns are clear, such as a high diversity of genes for amino acid uptake and a high diversity of ABC-type sugar transport genes in *Actinobacteria* and *Alphaproteobacteria*, coupled to a lack of genes for carboxylic acid uptake and a multitude of genes for polyamine uptake in *Actinobacteria*. The *Gammaproteobacteria*, *Bacteroidetes* and *Verrucomicrobia* encoded a large number of TonB-dependent transporter genes, likely involved in polymer uptake [137]. Phosphate uptake systems, such as the high affinity PstS transporter, were highly abundant in the betaproteobacterial BACL14, while *Thaumarchaeon* BACL13 had the highest proportion of phosphonate transporter genes, followed by the *Cyanobacterium*

BACL30 and the acIV genome clusters. BACL30 and the BACL13 were sparse in uptake systems for organic molecules in general, consistent with these organisms' photo- and chemoautotrophic lifestyles, respectively.

All *Bacteroidetes* and *Gammaproteobacteria* clusters contained the Na+-transporting NADH:ubiquinone oxidoreductase (NQR) enzyme gene. This enzyme is involved in the oxidative respiration pathway in some bacteria and is similar to the typical H+-transporting *ndh* NADH dehydrogenase [138]. However, the NQR enzyme exports sodium from the cell and thereby creates a gradient of Na+ ions, in contrast to the proton gradient generated by the *ndh* enzyme. The use of the NQR enzyme has been shown to be correlated with salinity (increasing Na+ concentrations) in bacterial communities [13]. Accordingly, NQR-containing MAG clusters were generally the ones with closest relatives in the marine environment (e.g. *Bacteroidetes*, see section on biogeography below), while genome clusters more closely related to freshwater bacteria (e.g. *Actinobacteria*) contained the H+-transporting enzyme. An exception to this were the SAR11 MAGs, which, like marine SAR11, harbored the H+-transporting enzyme. The genomes containing NQR enzymes in our dataset also contained a significantly higher proportion of Na+ symporters and antiporters (for e.g. dicarboxylates, disaccharides and amino-acids), as well as TonB-dependent transporters, compared to the other genomes (Welch's t-test $p<0.001$). In contrast, ATP-driven ABC-transporters were significantly less abundant in these clusters ($p<0.001$), strongly indicating that these bacteria have reduced their energy requirement by making use of the sodium motive force generated by the NQR enzyme to drive transport processes, a strategy that has been suggested previously [138]. TonB-dependent transporters require energy derived from charge separation across cellular membranes, generally in the form of a proton gradient [139]. The significant enrichment of TonB transporters in NQR-containing genomes suggests that these proteins may also utilize the sodium motive force.

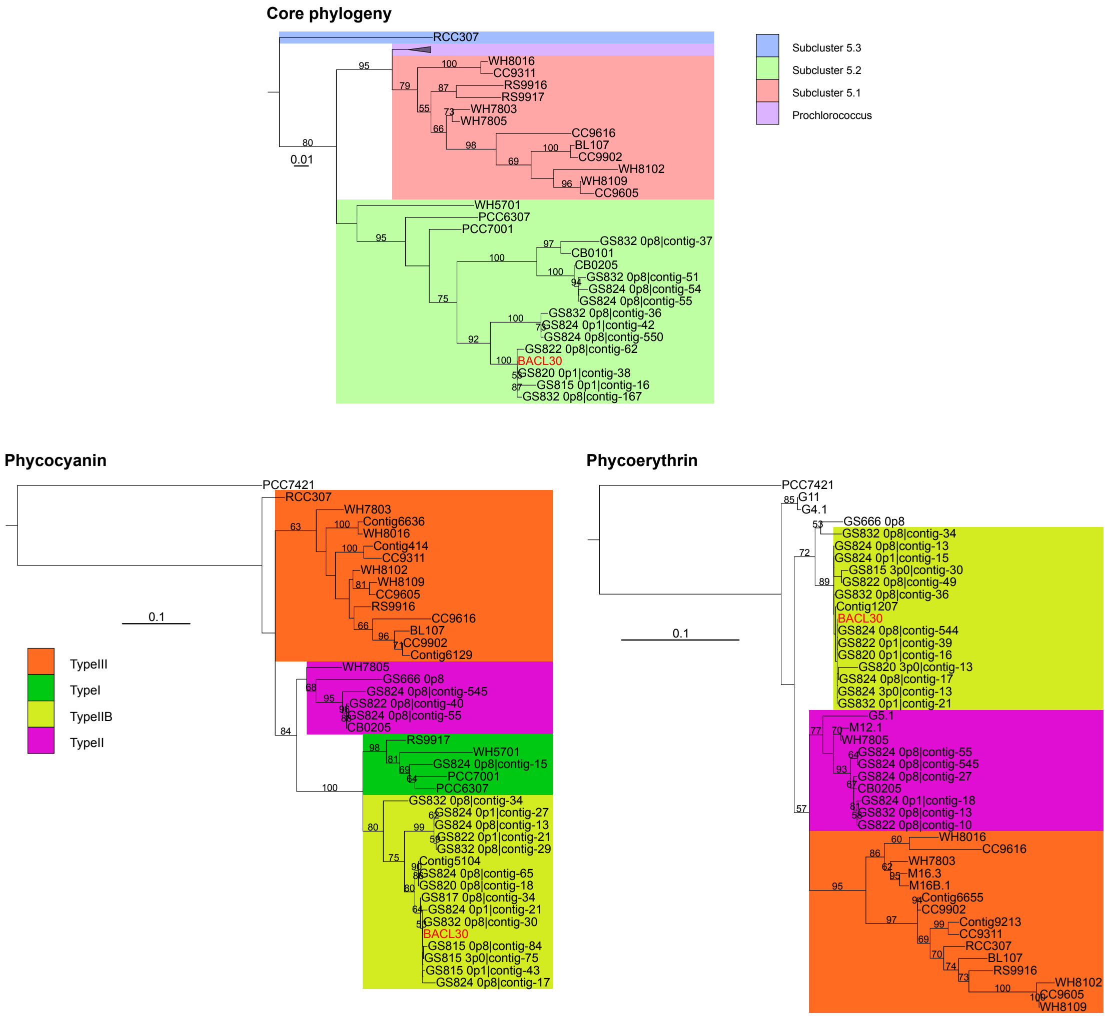

Figure S8. Core genome and pigment phylogeny of picocyanobacteria and BACL30. Phylogenetic trees are shown for core-genome proteins in picocyanobacteria (as in [57]) as well as concatenated alignments for phycocyanin (*cpcBA*) and phycoerythrin (*cpeBA*) gene products. In the core phylogeny, clade colors indicate subcluster designations for picocyanobacteria. In the pigment phylogenies, clade colors show pigment type designations. Scale bars indicate expected substitutions per site.

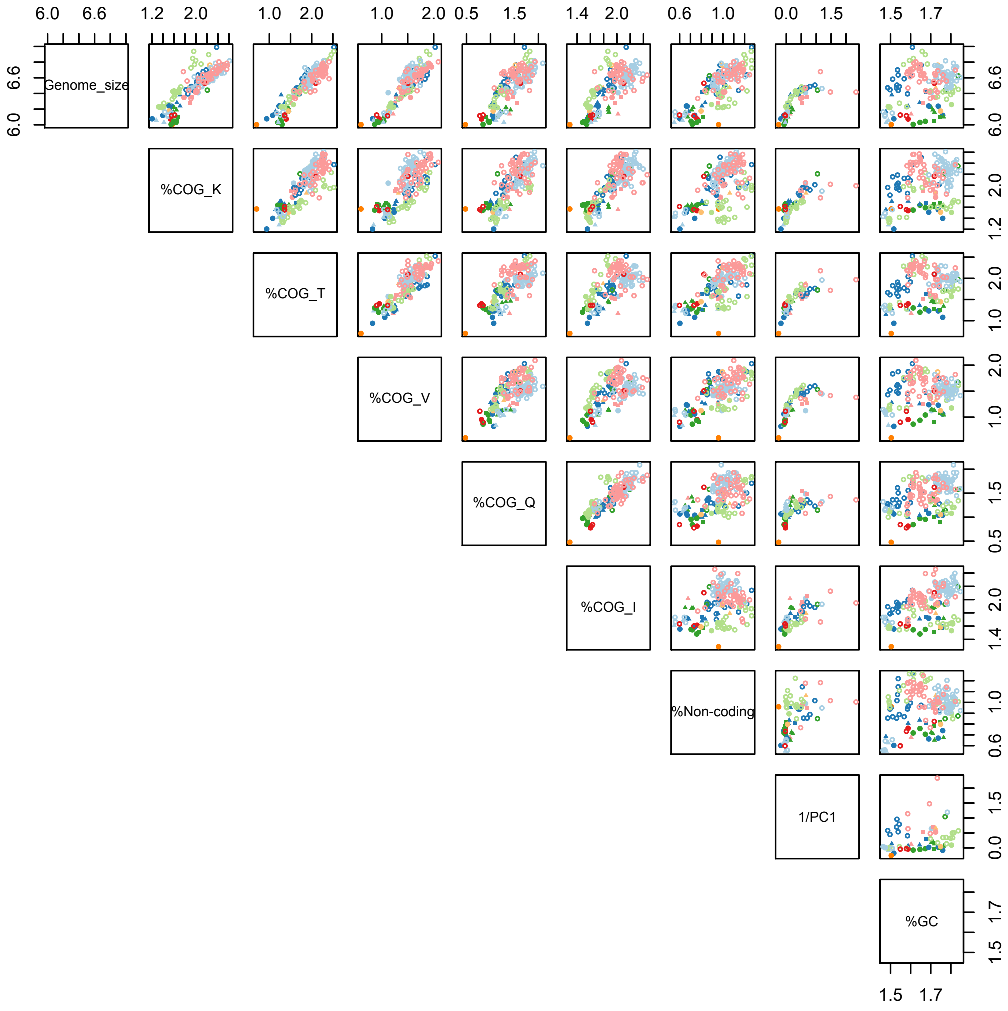

Figure S9. Pairwise scatter-plots showing correlations of genome features among MAGs and isolate genomes (all in log 10 scale). PC1 is the first principal component of the PCA of Figure 3a (that included all parameters except genome-size). Genomes are color-coded and shaped as in Figure 3.

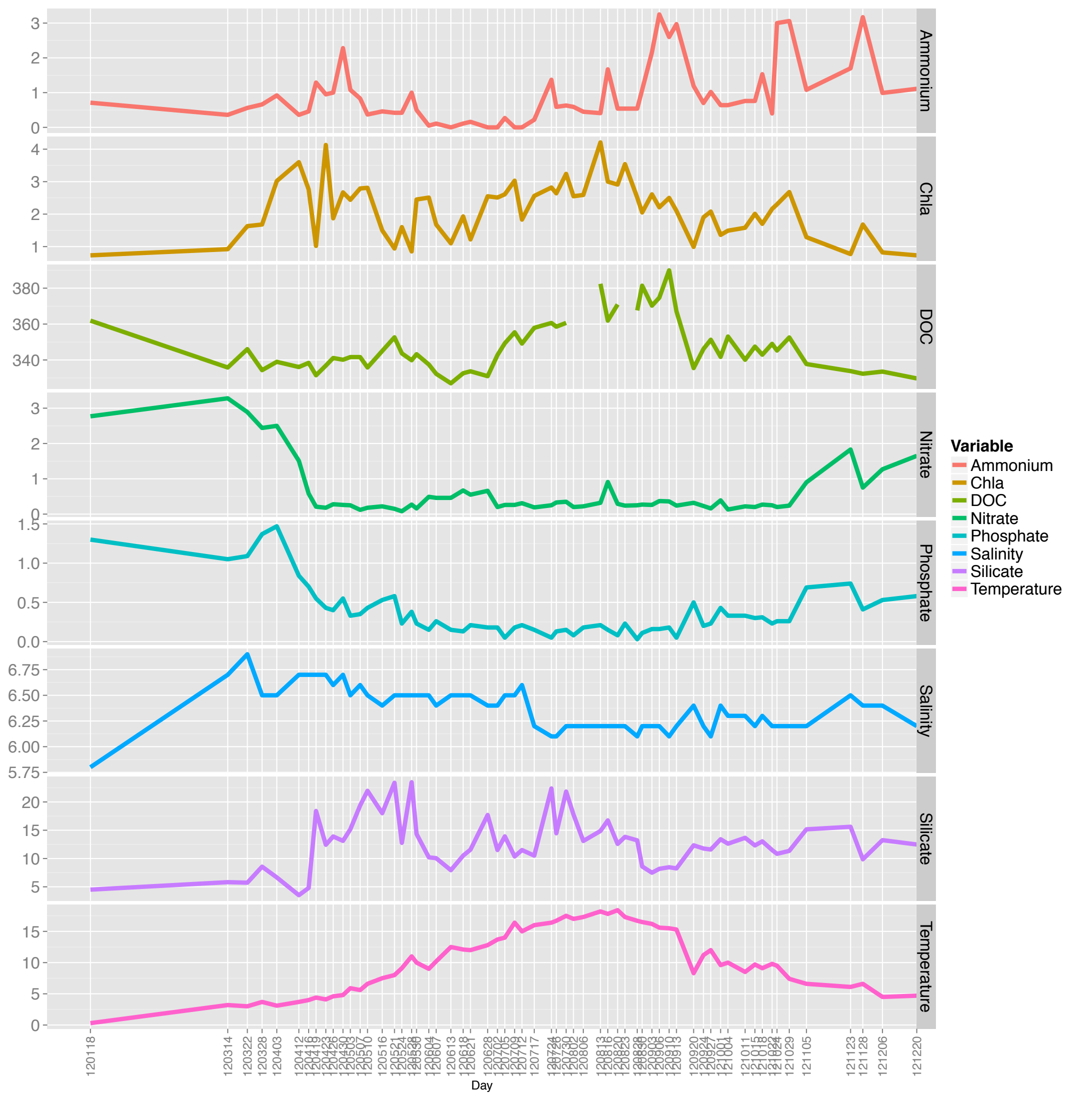

Figure S10. Variation in temperature (°C) and concentrations of nutrients and DOC (µM) and Chlorophyll a (µg/mL).

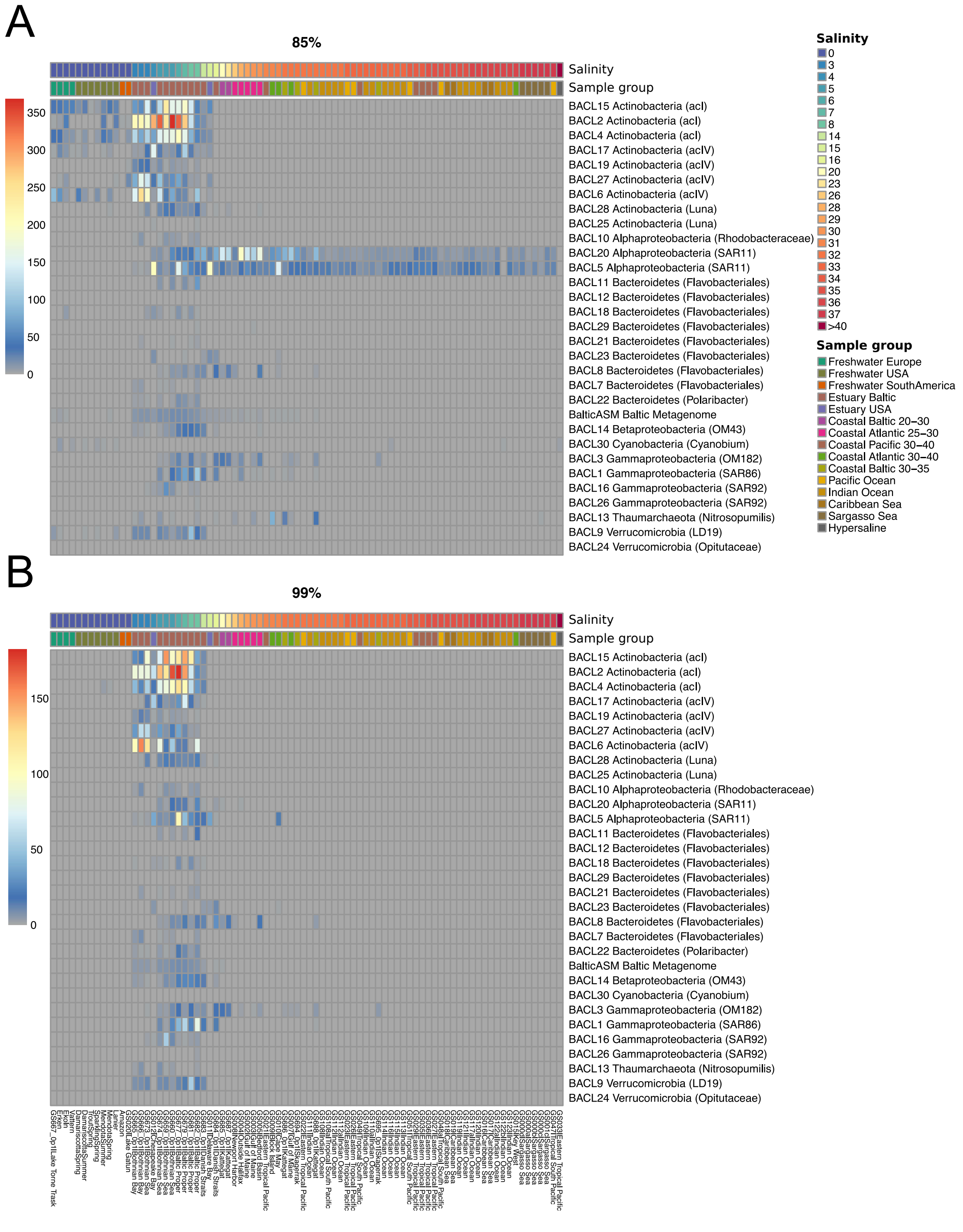

Figure S11. Biogeographical abundance profiles of MAGs. Heatmap plots showing the abundance of recruited reads from various samples and sample groups to each of the 30 MAG clusters at the (**a**) 85% and (**b**) 99% nucleotide identity cutoff levels. Shown values represent number of recruited reads/kb of genome per 10,000 queried reads. As in Figure 5 but with all samples in each sample group shown.

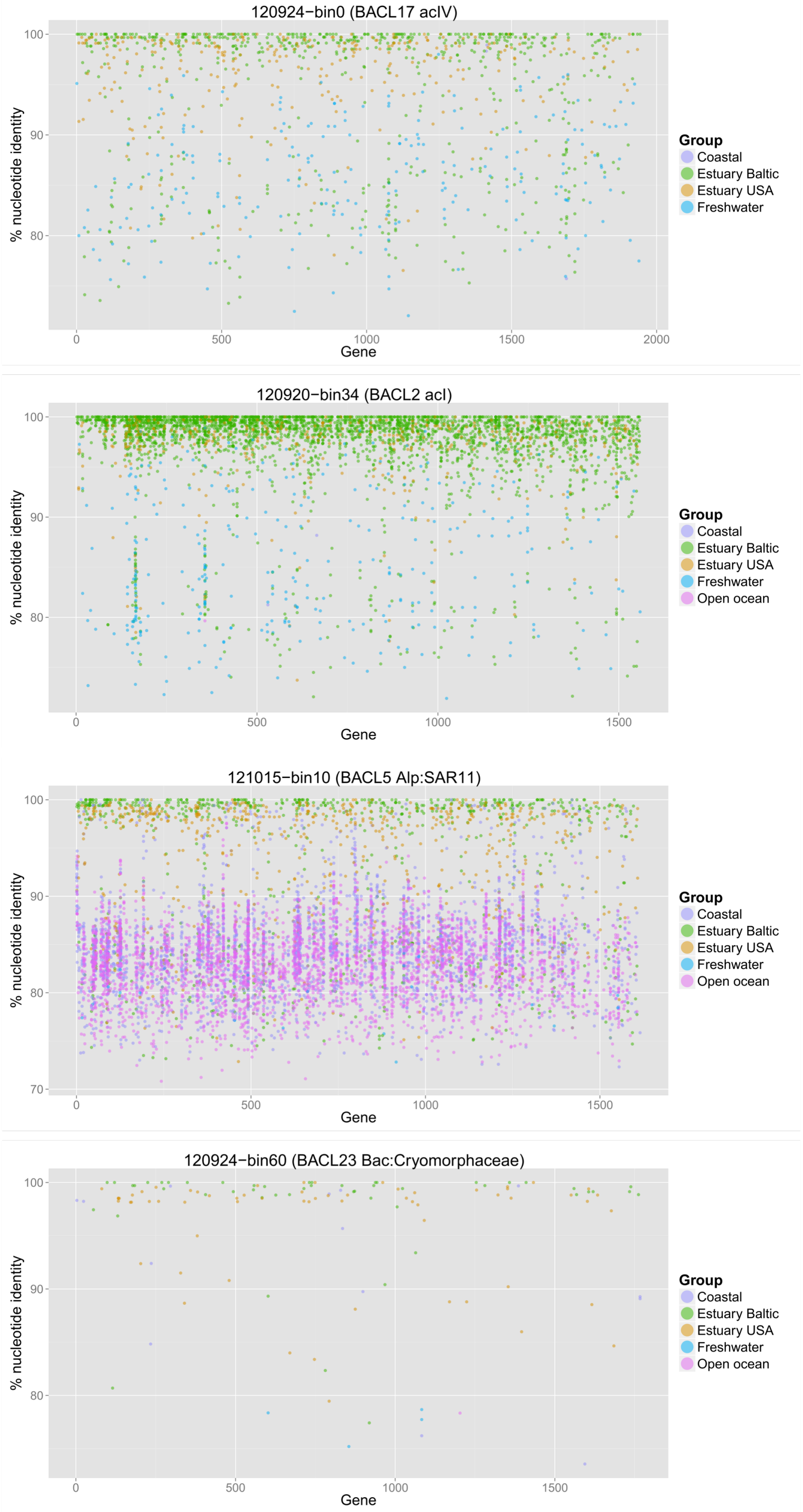

Figure S12. Fragment recruitment plots for selected MAG clusters. For each MAG cluster, the largest MAG is shown with the x-axis representing predicted open reading frames and the y-axis representing the nucleotide identity in %. Consequently, each recruited read is shown at a specific gene (x coordinate) and % identity (y coordinate) and further colored by sample group shown in the legend.

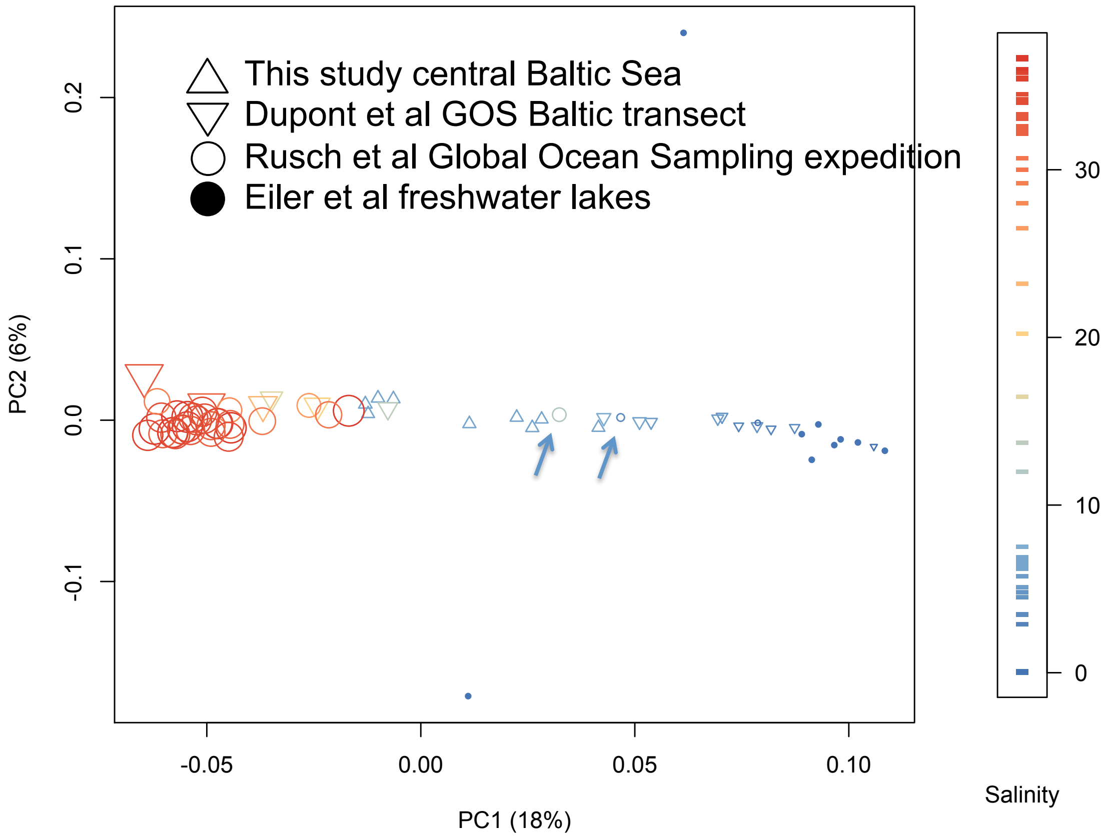

Figure S13. Principal coordinates analysis plot of aquatic metagenomes from different salinities. The salinity (range: 0 – 36.7 PSU) of the samples is indicated by size and color. We used 10 samples from this study representing different months, and samples from the Global Ocean Sampling expedition [12], the Baltic Sea GOS sampling [13], and metagenomic samples from freshwater lakes in Sweden and USA [109]. Note that the Dupont study included one lake sample (Torne Träsk) that groups with the lake samples from Eiler et al. It also includes marine samples from west of Sweden, grouping with the marine GOS samples from Rusch et al. The North American brackish GOS samples (Chesapeake Bay, salinity = 3.5 PSU; Delaware Bay, salinity = 15 PSU) are highlighted with arrows. The first principal coordinate (PC1) is highly correlated with salinity (Spearman rho = 0.89, p < 10-16). The Dupont samples were filtered with multiple filter sizes; here we used only the filter fraction 0.1 – 0.8 µm, but PC1 also correlated strongly with salinity when including the 0.8 – 3.0 µm fraction. 2164 of the 3880 detected COGs were significantly correlated with salinity (FDR adjusted Spearman correlation p-values < 0.05).

Annotation	Phylum-p	Phylum-R	Class-p	Class-R	Lineage-p	Lineage-R
**TIGRFAM**	0.001	0.874	0.001	0.966	0.001	0.973
**PFAM**	0.001	0.889	0.001	0.984	0.001	0.970
**COG**	0.001	0.868	0.001	0.967	0.001	0.977
**EC**	0.001	0.856	0.001	0.942	0.001	0.968
**Minpath**	0.001	0.785	0.001	0.840	0.001	0.984
**Transporters**	0.001	0.676	0.001	0.874	0.001	0.965

Table S1. Results from ANOSIM analysis of clustering of MAGs based on functional annotations. For all analyses, phyla, classes or lineages with only 1 representative were removed.

Supplementary references

114. Kang I, Lee K, Yang SJ, Choi A, Kang D, Lee YK, Cho JC: **Genome sequence of "Candidatus Aquiluna" sp. strain IMCC13023, a marine member of the Actinobacteria isolated from an arctic fjord**. *J Bacteriol* 2012, **194**:3550–3551.

115. Hahn MW, Schmidt J, Taipale SJ, Doolittle WF, Koll U: **Rhodoluna lacicola gen. nov., sp. nov., a planktonic freshwater bacterium with stream-lined genome**. *Int J Syst Evol Microbiol* 2014, **64**:3254–3263.

116. Garcia SL, McMahon KD, Martinez-Garcia M, Srivastava A, Sczyrba A, Stepanauskas R, Grossart H-P, Woyke T, Warnecke F: **Metabolic potential of a single cell belonging to one of the most abundant lineages in freshwater bacterioplankton**. *ISME J* 2013, **7**:137–147.

117. Glöckner FO, Zaichikov E, Belkova N, Denissova L, Pernthaler J, Pernthaler A, Amann R: **Comparative 16S rRNA Analysis of Lake Bacterioplankton Reveals Globally Distributed Phylogenetic Clusters Including an Abundant Group of Actinobacteria**. *Appl Environ Microbiol* 2000, **66**:5053–5065.

118. Newton RJ, Kent AD, Triplett EW, McMahon KD: **Microbial community dynamics in a humic lake: differential persistence of common freshwater phylotypes**. *Environ Microbiol* 2006, **8**:956–970.

119. Wu X, Xi W, Ye W, Yang H: **Bacterial community composition of a shallow hypertrophic freshwater lake in China, revealed by 16S rRNA gene sequences**. *FEMS Microbiol Ecol* 2007, **61**:85–96.

120. Humbert J-F, Dorigo U, Cecchi P, Le Berre B, Debroas D, Bouvy M: **Comparison of the structure and composition of bacterial communities from temperate and tropical freshwater ecosystems**. *Environ Microbiol* 2009, **11**:2339–2350.

121. Ghai R, McMahon KD, Rodriguez-Valera F: **Breaking a paradigm: cosmopolitan and abundant freshwater actinobacteria are low GC**. *Environ Microbiol Rep* 2012, **4**:29–35.

122. Bowman J: **The Family Cryomorphaceae**. In *The Prokaryotes*. Edited by Rosenberg E, DeLong E, Lory S, Stackebrandt E, Thompson F. Springer Berlin Heidelberg; 2014:539–550. [*135*]

123. Giovannoni SJ, Hayakawa DH, Tripp HJ, Stingl U, Givan SA, Cho J-C, Oh H-M, Kitner JB, Vergin KL, Rappé MS: **The small genome of an abundant coastal ocean methylotroph**. *Environ Microbiol* 2008, **10**:1771–1782.

124. Huggett MJ, Hayakawa DH, Rappé MS: **Genome sequence of strain HIMB624, a cultured representative from the OM43 clade of marine Betaproteobacteria**. *Stand Genomic Sci* 2012, **6**:11–20.

125. Kalyuzhnaya MG, Beck DAC, Vorobev A, Smalley N, Kunkel DD, Lidstrom ME, Chistoserdova L: **Novel methylotrophic isolates from lake sediment, description of Methylotenera versatilis sp. nov. and emended description of the genus Methylotenera**. *Int J Syst Evol Microbiol* 2012, **62**(Pt 1):106–111.

126. Crosbie ND, Pöckl M, Weisse T: **Dispersal and phylogenetic diversity of nonmarine picocyanobacteria, inferred from 16S rRNA gene and cpcBA-intergenic spacer sequence analyses**. *Appl Environ Microbiol* 2003, **69**:5716–5721.

127. Hugenholtz P, Pitulle C, Hershberger KL, Pace NR: **Novel division level bacterial diversity in a Yellowstone hot spring**. *J Bacteriol* 1998, **180**:366–376.

128. Zwart G, Huismans R, van Agterveld MP, Van de Peer Y, De Rijk P, Eenhoorn H, Muyzer G, van Hannen EJ, Gons HJ, Laanbroek HJ: **Divergent members of the bacterial division Verrucomicrobiales in a temperate freshwater lake**. *FEMS Microbiol Ecol* 1998, **25**:159–169.

129. Op den Camp HJ, Islam T, Stott MB, Harhangi HR, Hynes A, Schouten S, Jetten MS, Birkeland NK, Pol A, Dunfield PF: **Environmental, genomic and taxonomic perspectives on methanotrophic Verrucomicrobia**. *Environ Microbiol Rep* 2009, **1**.

130. Yoon J, Yasumoto-Hirose M, Katsuta A, Sekiguchi H, Matsuda S, Kasai H, Yokota A: **Coraliomargarita akajimensis gen. nov., sp. nov., a novel member of the phylum "Verrucomicrobia" isolated from seawater in Japan**. *IJSEM* 2007, **57**:959–963.

131. Karner MB, DeLong EF, Karl DM: **Archaeal dominance in the mesopelagic zone of the Pacific Ocean**. *Nature* 2001, **409**:507–510.

132. Wuchter C, Abbas B, Coolen MJL, Herfort L, van Bleijswijk J, Timmers P, Strous M, Teira E, Herndl GJ, Middelburg JJ, Schouten S, Sinninghe Damsté JS: **Archaeal nitrification in the ocean**. *Proc Natl Acad Sci U S A* 2006, **103**:12317–12322.

133. Walker CB, la Torrea JR de, Klotzc MG, Urakawaa H, Pinela N, Arpd DJ, Brochier-Armanete C, Chainf P, Chani PP, Gollabgirj A, Hempk J, Hüglerl M, Karrn EA, Könnekeo M, Shinf M, Lawtonp TJ, Lowei T, Martens-Habbenaa W, Sayavedra-Sotod LA, Langf D, Sievertq SM, Rosenzweigp AC, Manningj G, Stahla DA: **Nitrosopumilus maritimus genome reveals unique mechanisms for nitrification and autotrophy in globally distributed marine crenarchaea**. *Proc Natl Acad Sci U S A* 2010, **107**:8818–8823.

134. Park S, Ghai R, Martín-Cuadrado A, Rodríguez-Valera F, Chung W, Kwon K, Lee J, Madsen EL, Rhee S: **Genomes of Two New Ammonia-Oxidizing Archaea Enriched from Deep Marine Sediments**. *PLoS One* 2014:DOI: 10.1371/journal.pone.0096449.

135. Labrenz M, Sintes E, Toetzke F, Zumsteg A, Henrdl GJ, Seidler M, Jürgens K: **Relevance of a crenarchaeotal subcluster related to Candidatus Nitrosopumilus maritimus to ammonia oxidation in the suboxic zone of the central Baltic Sea**. *ISMEJ* 2010, **4**:1496–1508.

136. Alonso-Sáez L, Waller AS, Mende DR, Bakker K, Farnelid H, Yager PL, Lovejoy C, Tremblay J, Potvin M, Heinrich F, Estrada M, Riemann L, Bork P, Pedrós-Alió C, Bertilsson S: **Role for urea in nitrification by polar marine Archaea**. *Proc Natl Acad Sci U S A* 2012, **109**:17989–17994.

137. Tang K, Jiao N, Liu K, Zhang Y, Li S: **Distribution and functions of TonB-dependent transporters in marine bacteria and environments: implications for dissolved organic matter utilization**. *PLoS One* 2012, **7**:e41204.

138. Walsh DA, Lafontaine J, Grossart H-P: **On the Eco-Evolutionary Relationships of Fresh and Salt Water Bacteria and the Role of Gene Transfer in Their Adaptation**. In *Lateral Gene Transfer in Evolution*. Springer New York; 2013:55–77.

139. Noinaj N, Guillier M, Barnard TJ, Buchanan SK: **TonB-dependent transporters: regulation, structure, and function**. *Annu Rev Microbiol* 2010, **64**:43–60.
